# Taxonomy and multi-gene phylogeny of *Micropsalliota* (Agaricales, Agaricaceae) with description of six new species from China

**DOI:** 10.3389/fmicb.2022.1011794

**Published:** 2022-11-07

**Authors:** Jun-Qing Yan, Zhi-Heng Zeng, Ya-Ping Hu, Bin-Rong Ke, Hui Zeng, Sheng-Nan Wang

**Affiliations:** ^1^Jiangxi Key Laboratory for Conservation and Utilization of Fungal Resource, Jiangxi Agricultural University, Nanchang, China; ^2^Key Laboratory of State Forestry Administration on Forest Ecosystem Protection and Restoration of Poyang Lake Watershed, Jiangxi Agricultural University, Nanchang, China; ^3^Institute of Edible Mushroom, Fujian Academy of Agricultural Sciences, Fuzhou, China; ^4^National and Local Joint Engineering Research Center for Breeding and Cultivation of Features Edible Mushroom, Fuzhou, China; ^5^Nanjing Institute of Environmental Sciences, Ministry of Ecology and Environment, Nanjing, China; ^6^State Environmental Protection Scientific Observation and Research Station for Ecological Environment of Wuyi Mountains, Nanjing, China

**Keywords:** basidiomycete, biodiversity, East Asia, fungal phylogeny, new taxa, taxonomy

## Abstract

*Micropsalliota* is a relatively small genus containing only 62 previously identified species. Here, we describe six new taxa of *Micropsalliota* based on morphological and phylogenetic analyses: *M. minor, M. ovalispora, M. pseudodelicatula, M. rufosquarrosa, M. tenuipes*, and *M. wuyishanensis* and a new record taxon to China. The first Maximum likelihood and Bayesian analyses of a three-gene dataset (ITS, LSU, and *rpb2*) separated the genus into 18 weakly to strongly supported major clades and subclades, but only a few subclades were synapomorphies. According to phylogenetic analyses, *M. cornuta* does not belong in *Micropsalliota*. A key to 20 species of *Micropsalliota* in China is provided.

## Introduction

*Micropsalliota* Höhn. was circumscribed in 1914 based on the type species *M. pseudovolvulata* Höhn., which was collected from the Bogor Botanical Gardens, Indonesia, by the Austrian mycologist Höhnel in 1907 (Höhnel, 1914). It was once founded on inappropriate characters and considered a dubious genus. Later it was amended by Heinemann (1956) and Pegler and Rayner (1969), and accepted by Singer (1975). A molecular phylogenetic analysis based on ITS and LSU sequences has confirmed that *Micropsalliota* is monophyletic and sister to *Hymenagaricus* (Zhao et al., 2010). Species of *Micropsalliota* are characterized by the presence of usually small, gracile basidiomes with a membranous partial veil, dark spore prints, frequently capitate or subcapitate cheilocystidia, incrusted pileipellis hyphae that turn green in ammonia solution, and, in most species, basidiospores with an apically thickened endosporium and lacking a germ pore (Heinemann, 1977; Zhao et al., 2010).

*Micropsalliota* is not a very species-rich genus: only 79 names (62 species and five varieties), including synonyms, were listed in Index Fungorum (www.indexfungorum.org). Species of *Micropsalliota* are mainly distributed in tropical and subtropical Africa, America, and Asia (Heinemann, 1977, 1980, 1983, 1988, 1989; Heinemann and Flower, 1983; Heinemann and Leelavathy, 1991; Guzmán-Dávalos, 1992; Guzmán-Dávalos and Heinemann, 1994; Zhao et al., 2010; Chen et al., 2016; Parra et al., 2016; He et al., 2020; Crous et al., 2021; Al-Kharousi et al., 2022). Beginning with the discovery of *M. pseudoglobocystis* Li Wei and R. L. Zhao, 13 species, of which four are new, have been reported in China (Li et al., 2015, 2021; Wei et al., 2015; Wang et al., 2017; Chen et al., 2019; Sun et al., 2020; Liu et al., 2021). During our ongoing study of Chinese macrofungi initiated in 2018, the high species diversity of this genus in subtropical China has attracted our attention. In this paper, we present the results of molecular phylogenetic study based on ITS, LSU, and *rpb2* sequence datasets, examine the morphological synapomorphies of the different phylogenetic clades, and describe six new and a new recorded species from subtropical China based on morphological and molecular data.

## Materials and methods

### Morphological studies

Macroscopic descriptions and habitat details were based on detailed field notes of fresh basidiomata and photos. Color codes follow the Methuen Handbook of Color (Kornerup and Wanscher, 1978). Measuring stature follows the IG value (IG = St^2^/D × d, St = the length of the stipe, D = the diameter of the pileus, *d* = the diameter of the stipe): the corresponding value of slender is 50–150 and stout is 10–30 (Heinemann, 1983). Microscopic structures were observed and measured from dried specimens mounted in water, 5% KOH, 10% NH_4_OH, or Melzer's reagent. Congo red was used as a stain when necessary (Horak, 2005). A minimum of 80 basidiospores, 20 basidia, and 40 cystidia per specimen were randomly measured using an Olympus BX53 microscope. The measurements and Q values are recorded as (a)b–c(d), in which “a” is the lowest value, “b–c” covers a minimum of 90% of the values, and “d” is the highest value. Mean spore size is indicated by “av.”, and “Q” stands for the ratio of the length and width of a spore (Bas, 1969; Yu et al., 2020). Specimens were deposited in the Herbarium of Fungi, Jiangxi Agricultural University (HFJAU).

### DNA extraction and sequencing

DNA was extracted from dried specimens with the NuClean Plant Genomic DNA kit (CWBIO, China) (Ge et al., 2021; Na et al., 2022). Three regions (ITS, LSU, *rpb2*) were selected for the study and were amplified using the primer pairs ITS1/ITS4 (White et al., 1990), LR0R/LR7 (Hopple and Vilgalys, 1999), and 6F/7.1R (Matheny, 2005), respectively. PCR was performed using a touchdown program for all regions: 5 min at 95°C, 1 min at 95°C, 30 s at 65°C (adding −1°C per cycle), 1 min at 72°C, cycle 15 times; 1 min at 95°C, 30 s at 50°C, 1 min at 72°C, cycle 20 times; 10 min at 72°C (Bau and Yan, 2021). The sequencing was performed by Qing Ke Biotechnology Co. Ltd. (Wuhan City, China).

### Data analyses

All 240 nucleotide DNA sequences (ITS, LSU, and *rpb2*) of *Micropsalliota* in NCBI GenBank were downloaded. After discarding sequences shorter than 250 bp, 173 sequences were retained. In addition, 77 sequences were generated from our collected specimens. The *Agaricus crassisquamosus* R. L. Zhao, *A. trisulphuratus* Berk., *A. variicystis* Linda J. Chen, K. D. Hyde and R. L. Zhao, *Hymenagaricus epipastus* (Berk. and Broome) Heinem. and Little Flower, and *H*. sp. were chosen as outgroup taxa according to the results of Zhao et al. (2010) and Li et al. (2021). A total of 250 sequences (137 ITS, 84 LSU, 29 *rpb2*) representing 139 taxa were used in subsequent analyses. Details are presented in Supplementary Table S1.

ITS, LSU, and *rpb2* sequence datasets were separately aligned on the MAFFT online server using the E-INS-i option for ITS and the L-INS-i option for LSU and *rpb2* (Katoh et al., 2019). Bayesian inference (BI) and maximum likelihood (ML) phylogenetic analyses of the aligned concatenated dataset were carried out in MrBayes v.3.2.7a and IQTREE v.2.1.2, respectively (Nguyen et al., 2014) *via* the CIPRES web portal. For the BI analyses, optimal evolutionary models were selected using PartitionFinder2 (Lanfear et al., 2017) with the greedy algorithm and the AICc criterion. Four Monte Carlo Markov chains were run for 2 million generations, with the first 25% of trees discarded as burn-in (Ronquist et al., 2012). For the ML analysis, models of sequence evolution were assessed in IQ-Tree prior to the analysis. The ML analysis was conducted using the ultrafast bootstrap option with 1,000 replicates and allowing partitions to have different seeds (-spp). A nexus file, which is generated by Mesquite (Maddison 2008), contains an alignment sequence, and the original tree of ML and Bayes is deposited in Supplementary material 2.

## Results

### Phylogenetic analyses

A total of 2,645 characters were used in subsequent analyses (ITS, 764 bp; LSU, 1,230 bp; *rpb2*, 651 bp), of which 735 sites were variable and 590 were parsimony informative. The best-fit models used for the phylogenetic analyses were as follows: for the BI analysis, GTR + I + G for ITS, LSU; SYM + I + G for *rpb2* partitions; for the ML analysis, TVM + F + I + G4 for ITS, TN + F + I + G4 for LSU, and TNe + G4 for *rpb2*. The log-likelihood of the ML consensus tree was −14,153.009, and the average standard deviation of split frequencies was < 0.01 after 1,750,000 generations in the BI analysis. In the resulting trees, clades with a Bayesian posterior probability (BI-PP) ≥ 0.95 and ML bootstrap support (ML-BP) ≥ 75% were considered to be well-supported (Wang et al., 2022).

**Figure 1:**
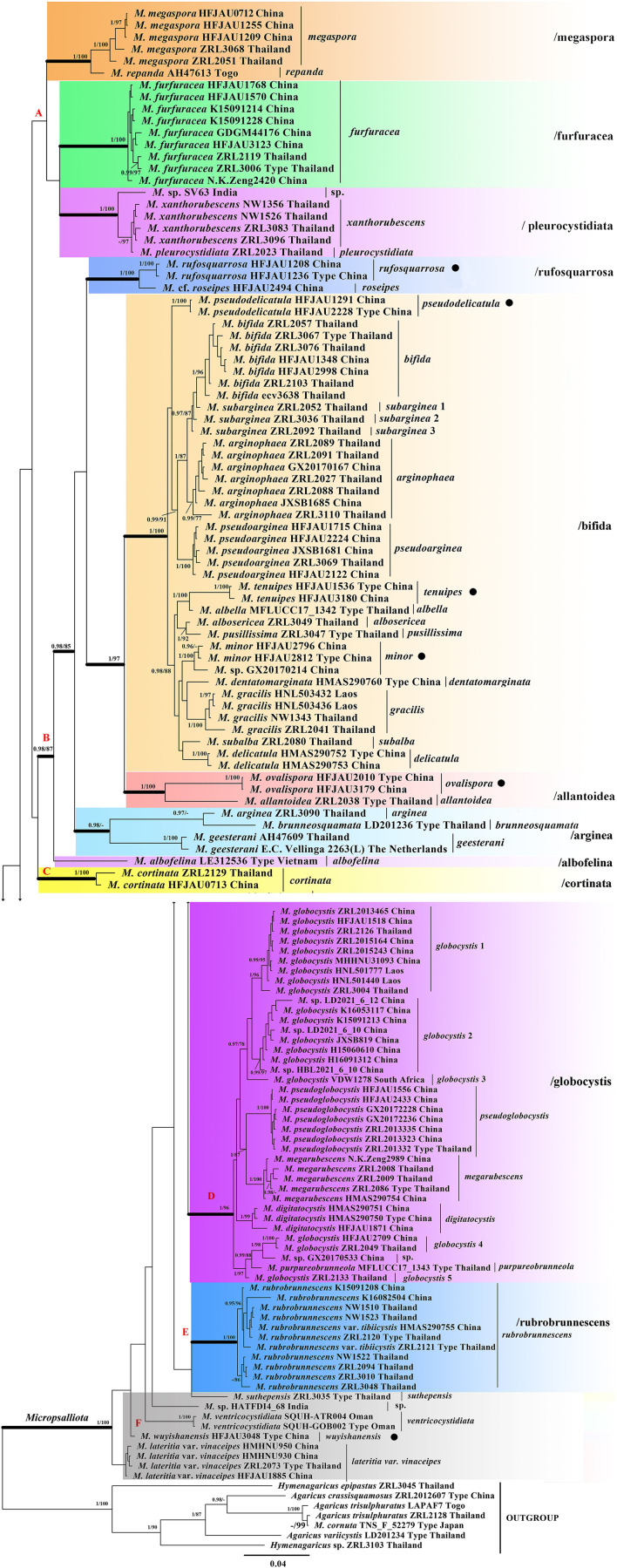
Phylogram of *Micropsalliota* generated by Bayesian inference (BI) analysis based on sequences of a concatenated data set from three nuclear genes (ITS, LSU, and *rpb2*), rooted with *Agaricus* spp. and *Hymenagaricus* spp. Posterior probabilities (BI-PP) ≥ 0.95 and ML bootstrap (ML-BP) ≥ 75% are shown as PP/BP. • indicates newly described taxa.

As shown in the phylogenetic tree in Figure 1, all taxa of *Micropsalliota*, except for *M. cornuta* Har. Takah. and Taneyama, formed a well-supported monophyletic lineage (BI-PP = 1; ML-BP = 100%). The type specimen of *M. cornuta* fell outside of *Micropsalliota* and formed a well-supported lineage with *A. trisulphuratus* collected from Togo (West Africa) and Thailand. The ITS sequences of these two species were 99% similar. The well-supported lineage closest to the root is taken as Clade. Four major lineages meet the definition (Clade B, C, D, E). Although Clade A does not form well-supported lineages, it contains only five species and forms a sister group with Clade B, so we also treat it as major Clade. Within *Micropsalliota*, in total, four Clade (Clade B, C, D, E) and 12 subclades (/albofelina, /allantoidea, /bifida, /furfuracea, /megaspore, /pleurocystidiata, /rufosquarrosa, and five distinct lineages represent independent species separately in Area F) were strongly supported, /arginea were only strongly supported in BI analyses and a major clade (Clade A) was weakly supported. They are described further below. Among the 20 species present in China, six were new taxa constituting individual lineages (BI-PP = 1; ML-BP = 100%) that were clearly distinct from closely related taxa.

Clade A was not supported but contained three well-supported subclades: /megaspore contained two species (*M. megaspora* R. L. Zhao, Desjardin, Soytong and K. D. Hyde and *M. repanda* Heinem.); /furfuracea consisted of a single species (*M. furfuracea* R. L. Zhao, Desjardin, Soytong and K. D. Hyde); /pleurocystidiata comprised *M. pleurocystidiata* Heinem. and Little Flower, *M. xanthorubescens* Heinem., and an unknown species.

Clade B was strongly supported (BI-PP = 0.98; ML-BP = 87%) and included five well-supported subclades: /rufosquarrosa comprised *M. rufosquarrosa* (new taxon) and *M*. cf. *roseipes* Heinem.; /bifida consisted of 14 species, of which three were new (*M. minor, M. pseudodelicatula*, and *M. tenuipes*) formed separate lineages and were clearly distinct. The three sequences of *M. subarginea* Heinem., all from Thailand, formed separate lineages, respectively; /allantoidea contained a new species (*M. ovalispora*) and a known species from Thailand (*M. allantoidea* R. L. Zhao, Desjardin, Soytong and K. D. Hyde), /arginea consisted of three species [*M. arginea* (Berk. and Broome) Pegler and R. W. Rayner, *M. brunneosquamata* Linda J. Chen, R. L. Zhao and K. D. Hyde, and *M. geesterani* (Bas and Heinem.) R. L. Zhao and L. A. Parra]; /albofelina consisted of a single species (*M. albofelina* D. D. Ivanova and O. V. Morozova).

Clade D was strongly supported (BI-PP = 1; ML-BP = 96%) and consisted of five known species (*M. globocystis* Heinem., *M. megarubescens* R. L. Zhao, Desjardin, Soytong and K. D. Hyde, *M. pseudoglobocystis, M. purpureobrunneola* M. Q. He and R. L. Zhao, and *M. digitatocystis* R. L. Zhao, J. X. Li and M. Q. He). But the sequences of *M. globocystis* from China and Thailand formed five distinct lineages.

Clade C and E contained only one species *M. cortinata* (Heinem.) Heinem. and *M. rubrobrunnescens* R. L. Zhao, Desjardin, Soytong and K. D. Hyde, separately. Area F consisted of five unaggregated lineages: the new species *M. wuyishanensis*, three known species (*M. lateritia* var. *vinaceipes* R. L. Zhao, Desjardin, Soytong & K. D. Hyde, *M*. *suthepensis* R. L. Zhao, Desjardin, Soytong and K. D. Hyde, and *M. ventricocystidiata* Al-Sadi and S. Hussain), and an undetermined specimen from India.

### Taxonomy

#### Micropsalliota minor J. Q. Yan sp. nov.

MycoBank: 842891

Etymology: Referring to its small basidiomata.

**Figure 2:**
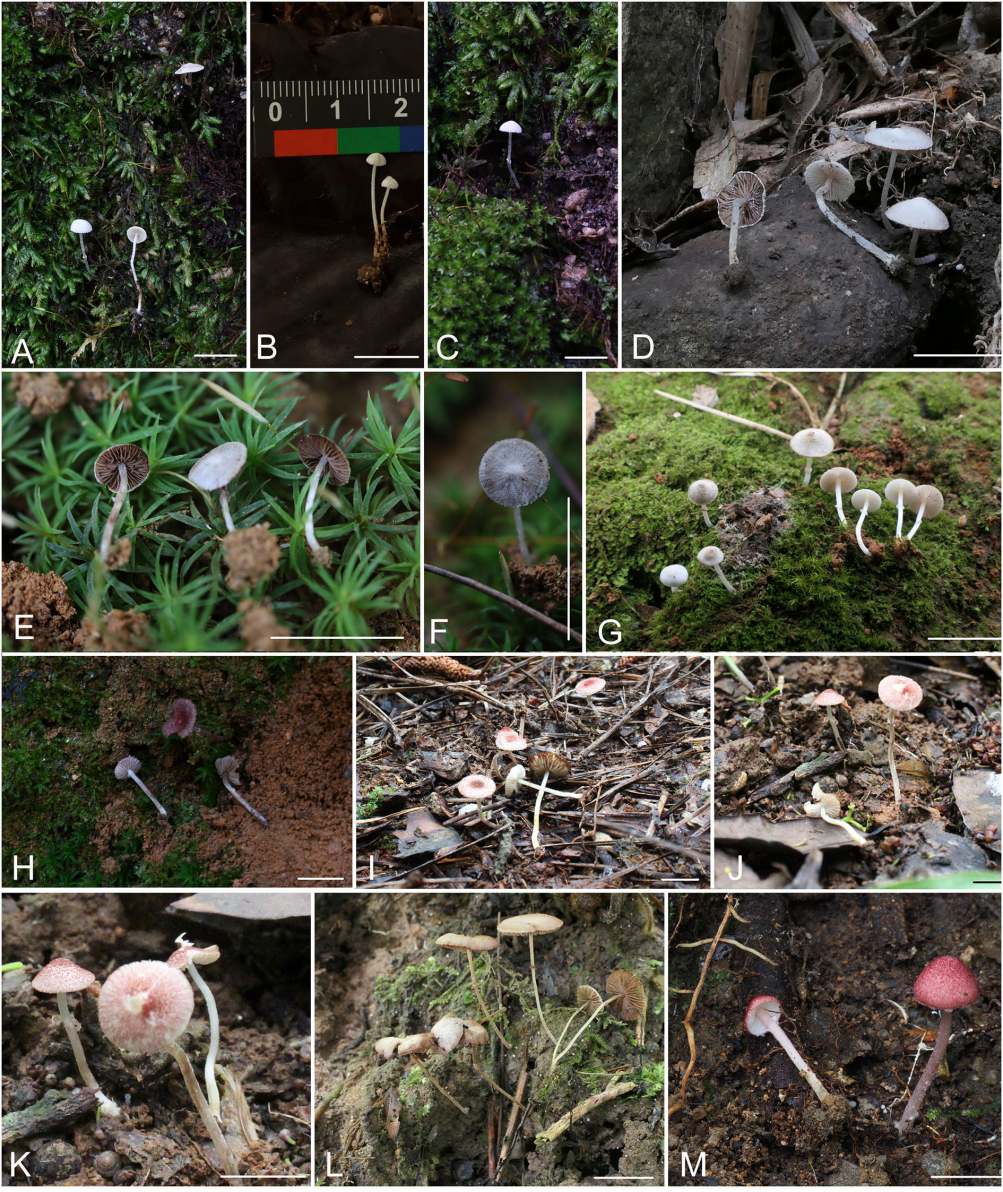
Basidiomata. **(A–C)** *Micropsalliota minor*; **(D)** *M. ovalispora*; **(E**–**G)** *M. pseudodelicatula*; **(H)** *M*. cf. *roseipes*; **(I–K)** *M. rufosquarrosa*; **(L)** *M. tenuipes*; **(M)** *M. wuyishanensis*. Scale bars: 10 mm.

**Figure 3:**
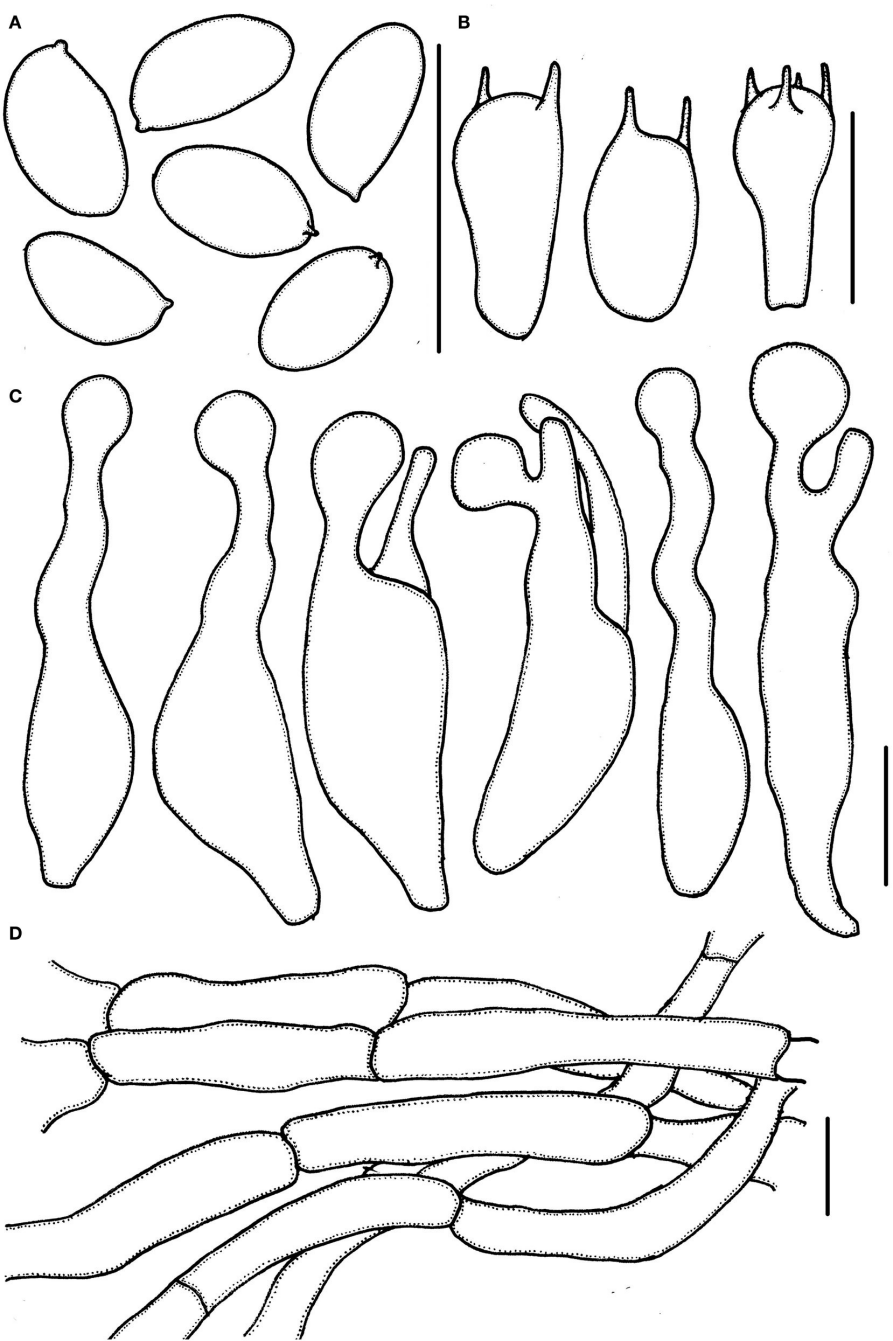
*Micropsalliota minor*. **(A)** Basidiospores; **(B)** basidia; **(C)** cheilocystidia; **(D)** pileipellis hyphae. Scale bars: 10 μm.

Diagnosis: Differs from *M. pusillissima* by bigger basidiospores, which are longer than 5 μm. (Figures 2A–C, 3, **10A**)

Basidiomata slender (IG = 40–192). Pileus 2.5–6.0 mm in diameter, white (1A1) to cream (2A1) when young, with light brown (6C6–6D6) tone in age, hemispherical, expanding to plano-convex, surface dry, glabrous to finely powdery fibrillose, margin appendiculate. Context less than 0.3 mm thick. Lamellae 0.2–0.5 mm broad, free, subdistant, with two series of lamellulae, white (1A1) becoming light brown (6C6–6D6) as mature. Stipe 12–17 × 0.3–0.6 mm, cylindrical, hollow, white (1A1), surface with white (1A1) fibrils, which easily fall off. Annulus single, membranous, median, white (1A1) to cream (2A1), which easily falls off.

Basidiospores (5.0) 5.5–6.0 (6.5) × 3.0–3.7 μm, av. = 5.7 × 3.4 μm, Q = (1.40) 1.50–1.82 (1.90), ellipsoid to elongated in face view, amygdaliform in profile view, light brown, wall 0.2–0.4 μm thick, apically thickened endosporium indistinct, without germ pore, inamyloid. Basidia 11–16 (17.5) × 5.5–6.5 (7.0) μm, clavate, hyaline, 2- or 4-spored. Pleurocystidia absent. Cheilocystidia (25) 29–45.5 (48) × (6.0) 6.5–11 μm, hyaline, tibiiform or lageniform, apex capitate, up to 4.0–6.0 μm in diameter or forked with capitate or obtuse to truncate apex. Pileipellis a cutis, hyphae 4.0–6.0 μm in diameter, hyaline, constricted at the septa on some hyphae.

Habit and habitat: Scattered on moss layer in mixed forests.

Specimens examined: CHINA. Zhejiang Province, Lishui City, Qingtian County, Huixu Village, 5 Aug 2021, Jun-Qing Yan, HFJAU2812, holotype; 6 Aug 2021, Jun-Qing Yan and Zhi-Heng Zeng, HFJAU2796; Jiangxi Province, Jiangxi Agricultural University, 3 Jun 2021, Jun-Qing Yan, HFJAU1259.

Notes: *Micropsalliota minor* formed an independent lineage in the phylogenetic tree. Morphologically, four *Micropsalliota* species (*M. alba* Heinem. and Little Flower, *M. albosericea* Heinem. and Leelav., *M. delicatula* R. L. Zhao, J. X. Li and M. Q. He, and *M. tenuipes*) have the same combination of small basidiomata with pileus generally less than 10 mm in diameter, white pileus, and basidiospores longer than 5.5 μm as *M. minor*, but none of them have the forked cheilocystidia (Heinemann and Flower, 1983; Zhao et al., 2010; Li et al., 2021). Another species, *M. bifida* R. L. Zhao, Desjardin, Soytong and K. D. Hyde, has variable (including forked) cheilocystidia, but its basidiospores are shorter than 5 μm (Zhao et al., 2010).

#### Micropsalliota ovalispora J. Q. Yan sp. nov.

MycoBank: 842892

Etymology: Referring to its oval basidiospores in face view.

**Figure 4:**
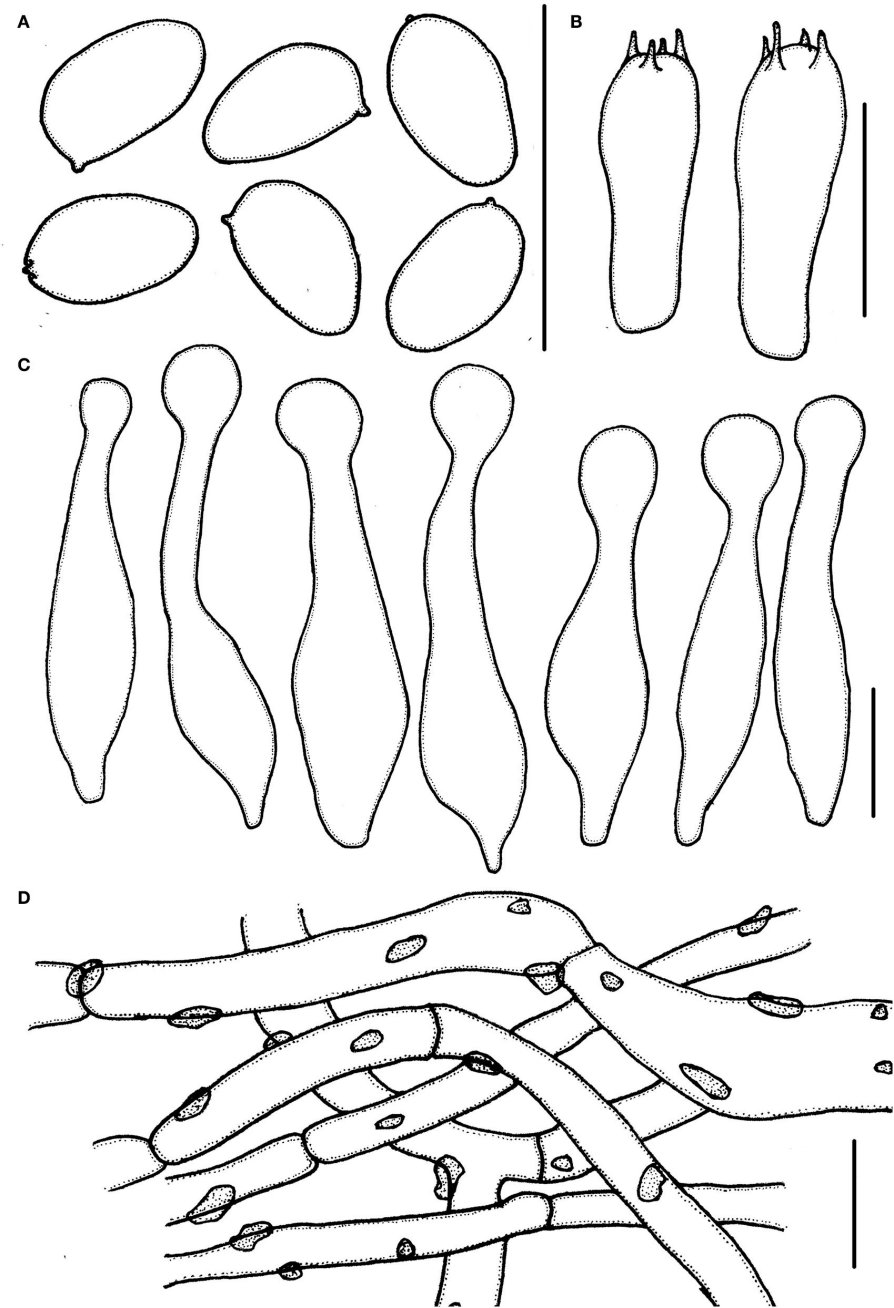
*Micropsalliota ovalispora*. **(A)** Basidiospores; **(B)** basidia; **(C)** cheilocystidia; **(D)** pileipellis hyphae. Scale bars: 10 μm.

Diagnosis: Differs from *M. arginea* in having ovoid basidiospores. (Figures 2D, 4, **10B**)

Basidiomata slender to stout (IG = 22–62). Pileus 5.0–7.0 mm in diameter, white (1A1), orange-gray (6B2) with age, convex, expanding to plane, with obtuse umbo, surface dry, glabrous to fibrillose. Context less than 0.5 mm thick. Lamellae 0.5–0.7 mm broad, free, moderately distant, with 2 series of lamellulae, white (1A1) becoming light brown (7D5–7D6) as mature. Stipe 7.5–12.5 × 0.5–1.0 mm, cylindrical, white (1A1), surface with white (1A1) fibrils. Annulus unobserved.

Basidiospores 4.0–5.0 × (2.0) 2.5–3.0 μm, av. = 4.5 × 2.7 μm, Q = (1.43) 1.55–1.85 (2.00), oval, rarely ellipsoid to elongate in face view, amygdaliform in profile view, light brown in water, darker in alkaline solution, wall 0.2–0.3 μm thick, apically thickened endosporium indistinct, without germ pore, inamyloid. Basidia 12.5–18.5 × 5.0–6.5 μm clavate, hyaline, 4-spored. Pleurocystidia absent. Cheilocystidia (22) 25.5–40 (42) × 4.0–8.5 (10.5) μm, lageniform, apex capitate, (4.0) 5.0–7.5 (8.0) in diameter. Pileipellis a cutis, hyphae 4.5–11 μm in diameter, constricted at the septa on some hyphae, with small dark brown incrusted pigment granules in water, which dissolves in 5% KOH.

Habit and habitat: Scattered on soil in broad-leaved forests.

Specimens examined: CHINA. Zhejiang Province, Lishui City, Suichang County, Jiulong Mountain Reserve, 15 Jul 2020, Jun-Qing Yan, HFJAU2010, holotype; 17 Jul 2020, Jun-Qing Yan and Bin-Rong Ke, HFJAU3179.

Notes: In the phylogenetic tree, *M. ovalispora* formed an independent lineage and grouped with *M. allantoidea*, but the latter has a grayish brown pileus and longer basidiospores (5.3–6.5 μm) (Zhao et al., 2010). Morphologically, only a few other species in the genus match the characteristics of *M. ovalispora*. *Micropsalliota arginea, M. cinnamomeopallida* Singer, *M. plumeria* (Berk. and Broome) Höhn., *M. pseudoarginea* Heinem., and *M. tenuipes* are similar to *M. ovalispora* in having a white pileus and 5.0–10 μm in diameter, but none of them have oval basidiospores (Pegler and Rayner, 1969; Heinemann, 1980; Heinemann and Leelavathy, 1991; Zhao et al., 2010). In addition, *M. arginea, M. pseudoarginea, M. ovalispora*, and *M. tenuipes* are in distinct lineages in the phylogenetic tree (this paper); pileipellis of *M. cinnamomeopallida* has brownish vacuolar pigment (Heinemann, 1980; Heinemann and Leelavathy, 1991); pileus of *M. plumeria* has rusty brown disc (Zhao et al., 2010).

#### Micropsalliota pseudodelicatula J. Q. Yan sp. nov.

MycoBank: 842893

Etymology: Referring to its morphological similarity to *M. delicatula*.

**Figure 5:**
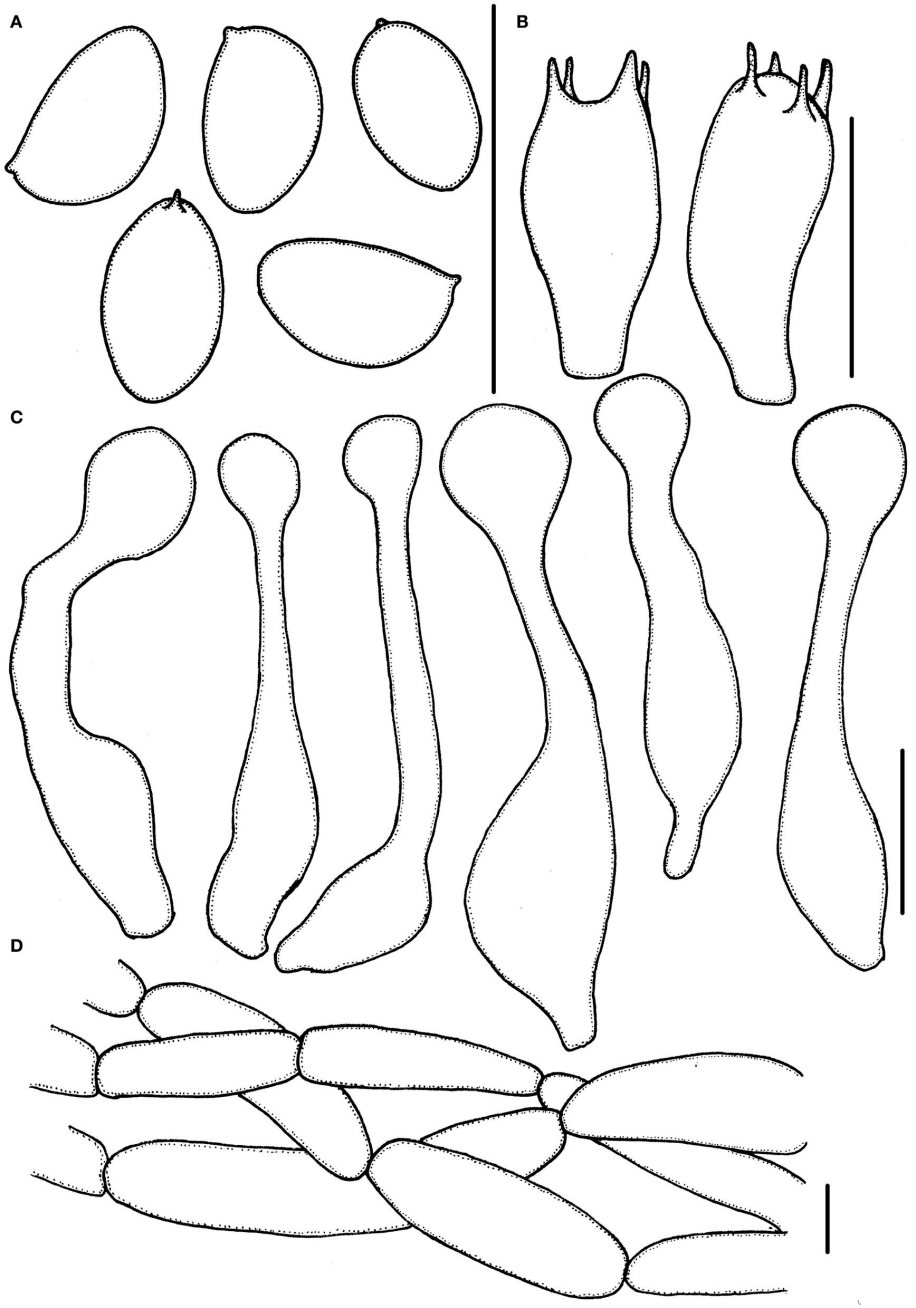
*Micropsalliota pseudodelicatula*. **(A)** Basidiospores; **(B)** basidia; **(C)** cheilocystidia; **(D)** pileipellis hyphae. Scale bars: 10 μm.

Diagnosis: Differs from *M. delicatula* in having smaller basidiospores (4.3–5.5 × 2.7–3.3 μm). (Figures 2E–G, 5, **10C**)

Basidiomata slender (IG = 50–93). Pileus 4.0–5.0 mm in diameter, white (1A1), convex to applanate, then nearly plane, surface dry, covered with white (1A1) scurfy scales or fibrils. Context less than 0.5 mm thick. Lamellae 0.5–1.0 mm broad, free, subdistant, with 2 series of lamellulae, white (1A1) becoming grayish red (7B2–7B3) to chestnut-brown (8D4–8E5) as mature, edge white (1A1). Stipe 8.0–15 × 0.3–1.0 mm, cylindrical, slender, hollow, white (1A1), surface with white (1A1) fibrils, base unexpanded, or slightly expanded to the shape of suction cup. Annulus single, membranous, persistent, superior to medium, white (1A1).

Basidiospores (4.0) 4.3–5.5 × (2.5) 2.7–3.3 μm, av. = 4.9 × 3.0 μm, Q = (1.39) 1.48–1.83 (1.95), ellipsoid to elongated in face view, amygdaliform in profile view, light brown, wall 0.2–0.4 μm thick, apically thickened endosporium indistinct, without germ pore, inamyloid. Basidia 11–14 × 3.5–6.0 μm, clavate, hyaline, 4- or 2-spored. Pleurocystidia absent. Cheilocystidia 29–40 (43) × (4.0) 5.0–8.5 (9) μm, often in clusters, tibiiform, few lageniform, apex capitate 6.0–9.0 μm in diameter. Pileipellis a cutis, hyphae 6.0–15 μm in diameter, hyaline, constricted at the septa on some hyphae.

Habit and habitat: Gregarious on red soil in forest.

Specimens examined: CHINA. Zhejiang Province, Suichang County, Sanren She nationality township, 29 May 2020, Jun-Qing Yan and Qin Na, HFJAU2228, holotype; Jiangxi Province, Jiangxi Agricultural University, 26 Jun 2019, Jun-Qing Yan, HFJAU1291.

Notes: *Micropsalliota pseudodelicatula*, which forms an independent lineage in subclade /bifida, is easily confused with *M. delicatula*. However, *M. delicatula* has larger basidispores, 5.2–6.6 × 3.0–4.1 μm (Li et al., 2021). In addition, *M. alba, M. albella* M. Q. He and R. L. Zhao, *M*. *albosericea, M. minor, M. pulverulenta* Heinem. and Leelav., and *M. pusillissima* R. L. Zhao, Desjardin, Soytong and K. D. Hyde all have tiny basidiomata (pileus 5 mm in diameter or less) that are similar to *M. pseudodelicatula*. These species can be distinguished as follows: basidiospores of *M. alba* are larger, up to 5.8–6.6 × 3.3–3.6 μm (Heinemann and Flower, 1983); *M. albella* has narrowly utriform to utriform cheilocystidia, some of which are slightly subcapitate (He et al., 2020); cheilocystidia of *M*. *albosericea* are smaller (18–30 × 6.0–11 μm) and clavate to ventricose-capitate or subcapitate, without obviously elongated neck (Zhao et al., 2010); *M. minor* has forked cheilocystidia (this paper); *M. pulverulenta* has pleurocystidia (Heinemann and Leelavathy, 1991); *M. pusillissima* has smaller basidiomata (1.0–3.0 mm) and possesses broadly ventricose-capitate cheilocystidia without an obviously elongated neck (Zhao et al., 2010).

#### Micropsalliota cf. Roseipes Heinem., Bull. Jard. Bot. natn. Belg. 50(1-2): 65 (1980) (Figures 2H, 6, **10D**)

Basidiomata stout (IG = 15–38). Pileus 6.0–10 mm in diameter, dirty white (1A1) to pink (11A5–11A6), expanding to plane, plane with aged, covered with dull red (11B4–11B5) to violet-red (11E7–11E8) squamules. Context less than 0.5 mm thick. Lamellae 0.5–0.1 mm broad, free, subdistant, with 2–3 series of lamellulae, white becoming light brown (6C6–6D6) as mature. Stipe 8.5–11.5 × 0.5–1.0 mm, cylindrical, dirty white (1A1) with some pinkish (11A5–11B5) tone, surface with white (1A1) fibrils. Annulus single, membranous, superior, white (1A1), easily fall off.

**Figure 6:**
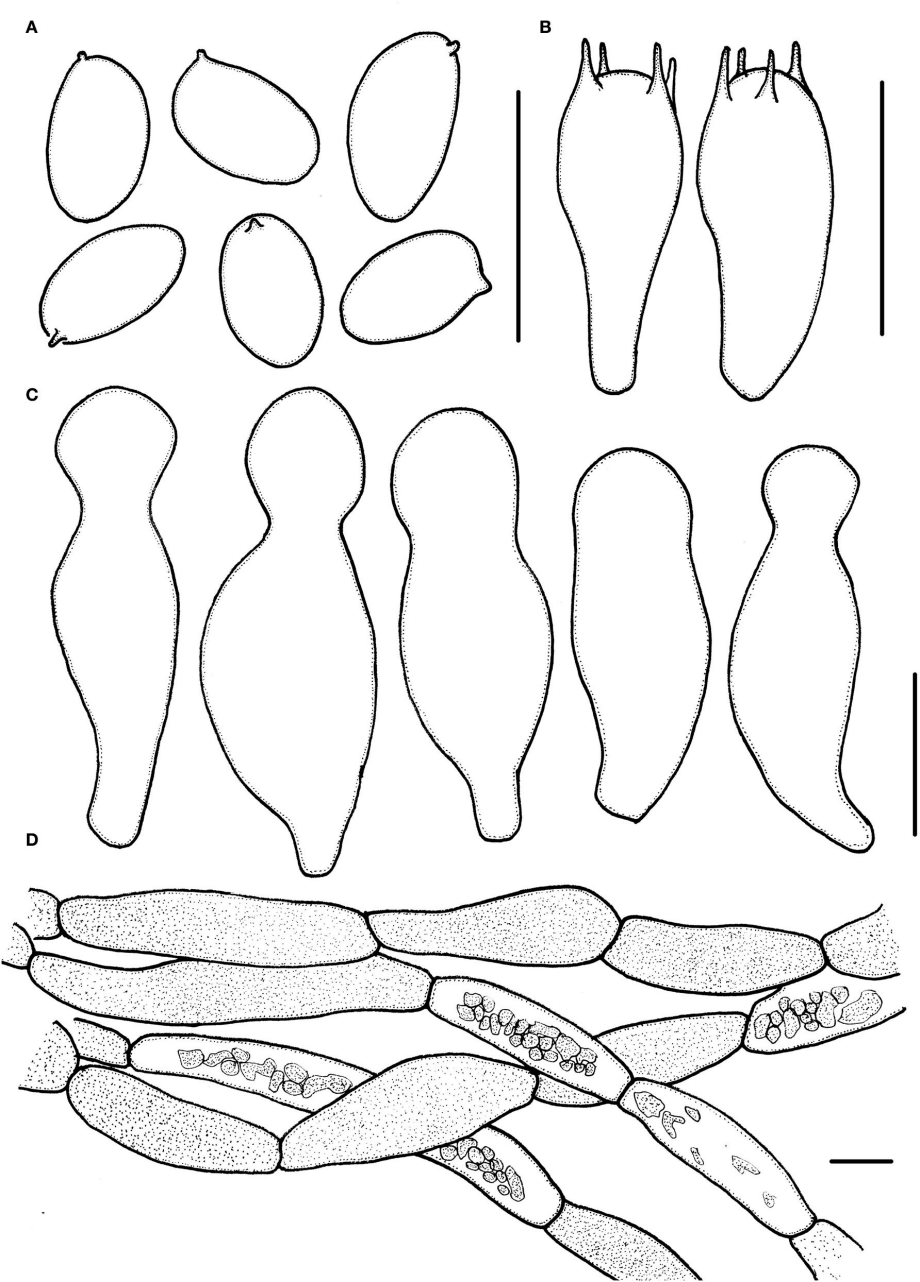
*Micropsalliota* cf. *roseipes*. **(A)** Basidiospores; **(B)** basidia; **(C)** cheilocystidia; **(D)** pileipellis hyphae. Scale bars: 10 μm.

Basidiospores 5.5–7.0 × 3.0–4.0 μm, av. = 6.2 × 3.6 μm, Q = 1.60–1.90 (2.00), elongated, rarely ovoid in face view, amygdaliform in profile view, light brown, wall 0.2–0.3 μm thick, apically thickened endosporium indistinct, without germ pore, inamyloid. Basidia 15.5–22.5 × 4.5–6.0 μm, clavate, hyaline, 4-spored. Pleurocystidia absent. Cheilocystidia 19–29 (34) × 6.5–9.0 (9.5) μm, utriform with obtuse, subcapitate or capitate apex, 5.0–7.5 μm in diameter. Pileipellis a cutis, hyphae (6.5) 8.0–18 (22) μm in diameter, constricted at the septa on some hyphae, with pink membranous pigment, or dark brown granular-incrusted in cellular vacuoles which can dissolve in 5% KOH (vacuolar pigment) in some hyphae.

Habit and habitat: Scattered on red soil in mixed forests.

Specimen examined: CHINA. Fujian Province, Wuyishan National Park, 20 June 2021, Jun-Qing Yan and Cheng-Feng Nie, HFJAU2494.

Notes: Chinese material closely matches the description of the type of *M. roseipes*, only differs in having narrower basidiospores, 3.0–4.0 μm rather than 4.0–4.8 μm (Heinemann, 1980). *Micropsalliota roseipes* is similar to *M. cardinalis* Heinem., *M. rufosquarrosa* and *M. wuyishanensis*, and groups together with *M. rufosquarrosa* in the phylogenetic tree; however, *M. cardinalis* has narrowly utriform cheilocystidia (Heinemann, 1989), *M. rufosquarrosa* has a white stipe and cheilocystidia covered by light brown deposition, and *M. wuyishanensis* has hyphoid and frequently forked cheilocystidia (this paper).

#### Micropsalliota rufosquarrosa J. Q. Yan sp. nov.

MycoBank: 842896

Etymology: Referring to its dull red squarrose pileus.

**Figure 7:**
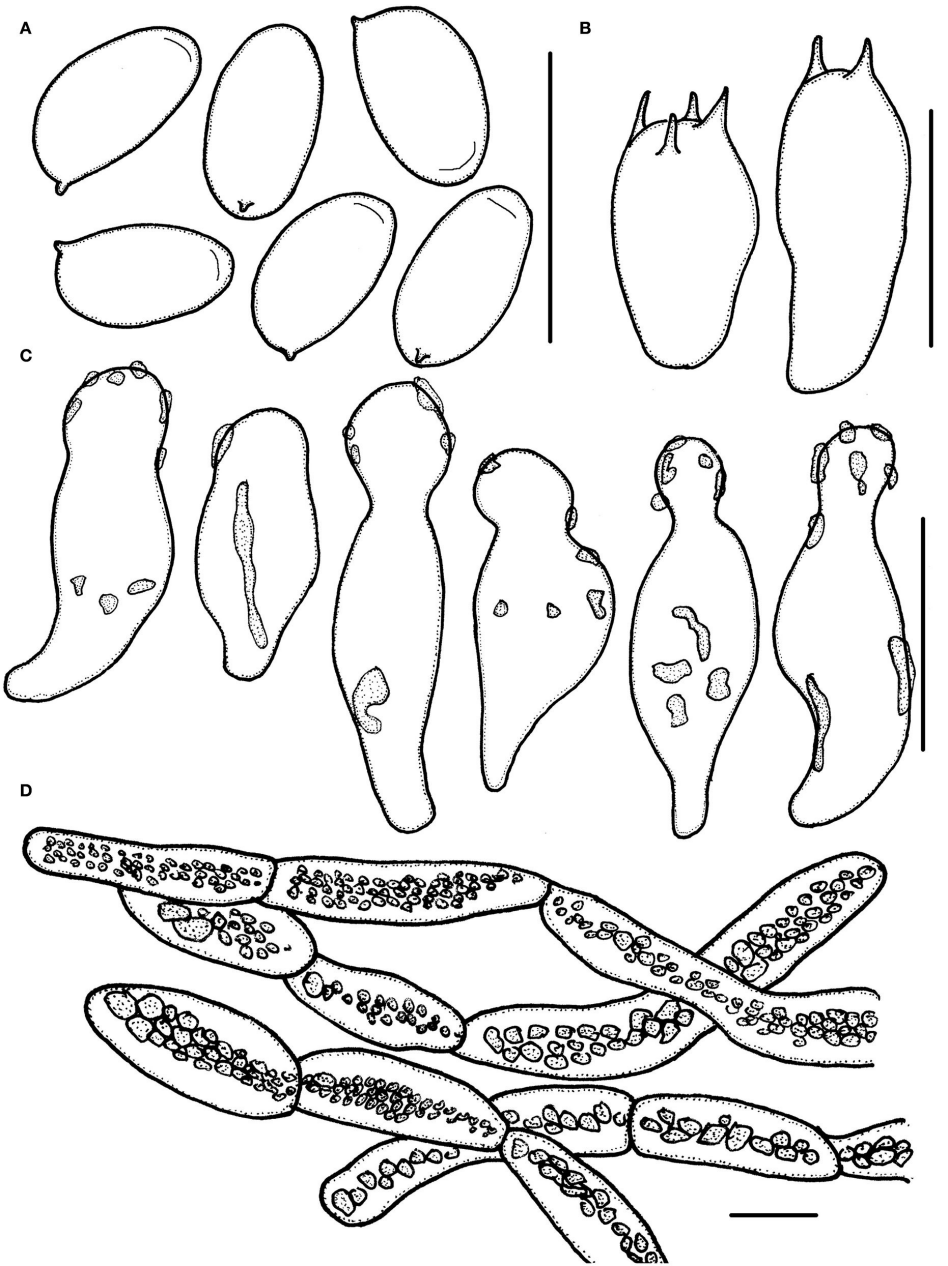
*Micropsalliota rufosquarrosa*. **(A)** Basidiospores; **(B)** basidia; **(C)** cheilocystidia; **(D)** pileipellis hyphae. Scale bars: 10 μm.

Diagnosis: Differs from *M. gracilis* in having smaller basidiomata and smaller cheilocystidia covered by an obvious brown deposition. (Figures 2I–K, 7, **10E**)

Basidiomata stout (IG = 9.0–33). Pileus 6.0–12 mm in diameter, white to dirty white (1A1–1B1), convex, expanding to plane, surface dry, covered with red (11B7) to brownish violet (11C7–11D7) squarrose. Context less than 0.5 mm thick. Lamellae 0.3–1.5 mm broad, free, subdistant, with 2 series of lamellulae, white becoming light brown (7D5–7D6) as mature. Stipe 18–38 × 0.5–1.5 mm, cylindrical, white (1A1), surface with white (1A1) fibrils. Annulus single, membranous, superior, white, with a reddish margin, easily fall off.

Basidiospores 5.5–6.5 (7.0) × 3.0–3.5 (4.0) μm, av. = 6.1 × 3.3 μm, Q = (1.60) 1.65–2.00 (2.25), elongated in face view, amygdaliform in profile view, light brown, wall 0.3–0.4 μm thick, most basidiospore with an apically thickened endosporium, without germ pore, inamyloid. Basidia 12.5–16.5 × 5.5–7.0 (7.5) μm, clavate, hyaline, 4- or 2-spored. Pleurocystidia absent. Cheilocystidia 14–26 × 6.0–10 μm, hyaline, utriform, apex broadly obtuse or capitate, 4.0–6.5 μm in diameter, covered by light brown deposition. Pileipellis a cutis, hyphae 4.5–14 (17) μm in diameter, constricted at the septa on some hyphae, with dark brown granular-incrusted in cellular vacuoles that dissolves in alkaline solution (vacuolar pigment).

Habit and habitat: Scattered on ground in mixed forests.

Specimens examined: CHINA. Jiangxi Province, Jiangxi Agricultural University, 20 May 2019, Jun-Qing Yan, HFJAU1208; 26 May 2019, Jun-Qing Yan, HFJAU1236, holotype.

Notes: Few species in the genus match the combination of small basidiomata, a pileus covered with dull red scurfy scales or fibrils, and small cheilocystidia covered by light brown deposition that characterizes *M. rufosquarrosa*. Some species have small basidiomata and a colored pileus but can be separated as follows: *M. allantoidea* has grayish brown scaly pileus, utriform, lageniform, or tiibiform capitate or subcapitate cheilocystidia (Zhao et al., 2010); *M. atropurpurea* Heinem., *M. cymbispora* Heinem. and Little Flower, and *M. pseudovolvulata* have smaller basidiospores averaging less than 5.5 μm long (Heinemann and Flower, 1983; Heinemann, 1988; Zhao et al., 2010); *M. cardinalis* and *M. purpureobrunneola* have purple to brownish purple scales and different cheilocystidia (Heinemann, 1989; He et al., 2020); *M. endophaea* Heinem., *M. megaspora*, and *M. roseipes* have larger basidiospores that are longer than 6.5 μm on average (Heinemann, 1988; Zhao et al., 2010); *M. malabarensis* Heinem. and Little Flower and *M. subalpina* Guzm.-Dáv. and Heinem. have longer cheilocystidia, up to 60 μm (Heinemann and Flower, 1983; Guzmán-Dávalos and Heinemann, 1994); *M. pruinosa* Heinem. has grayish brown scaly pileus, and ellipsoid spores in face view (Heinemann, 1989).

#### Micropsalliota tenuipes J. Q. Yan sp. nov.

MycoBank: 842897

Etymology: Referring to its slender stipe.

**Figure 8:**
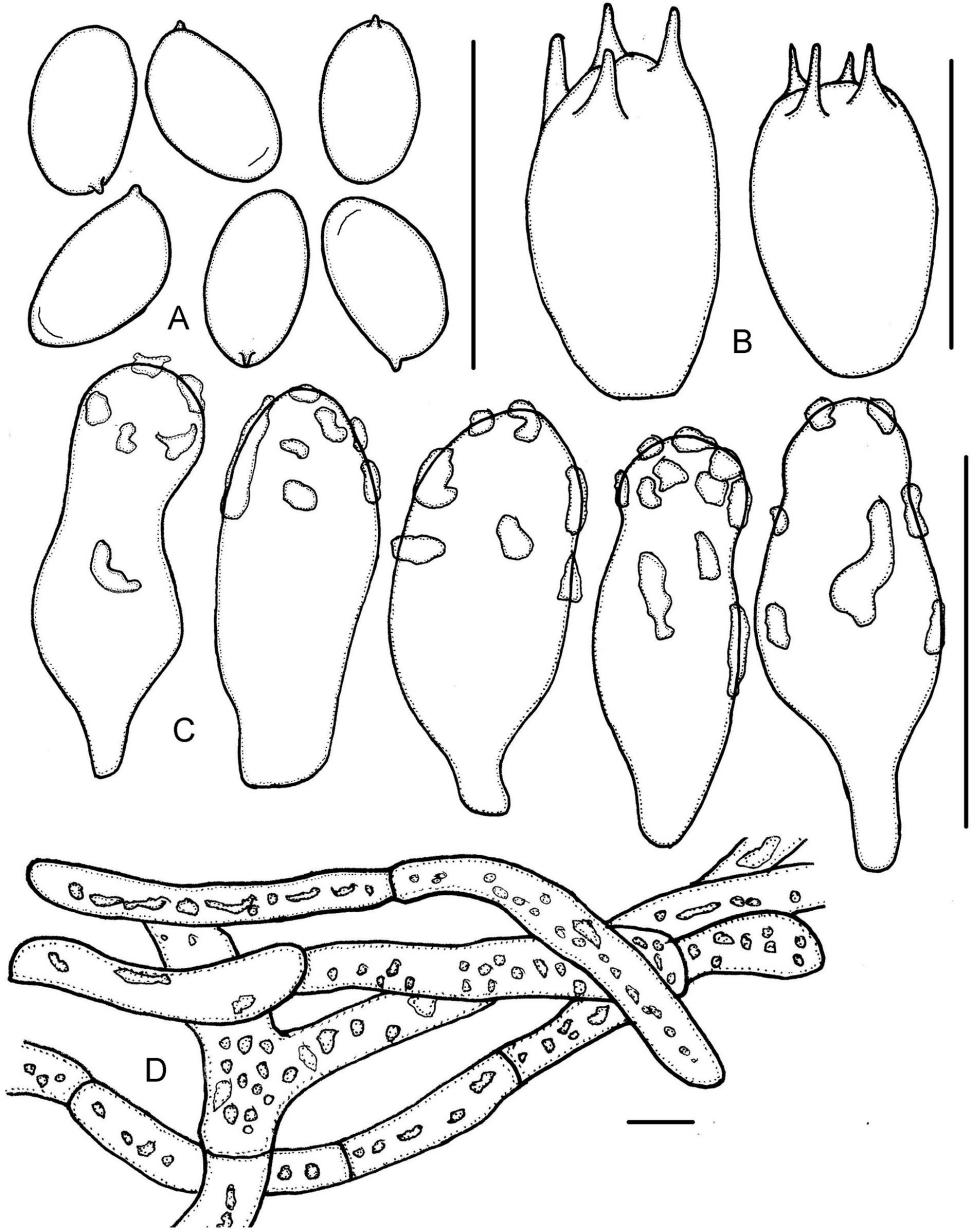
*Micropsalliota tenuipes*. **(A)** Basidiospores; **(B)** basidia; **(C)** cheilocystidia; **(D)** pileipellis hyphae. Scale bars: 10 μm.

Diagnosis: Differs from *M. arginea* in having bigger basidiospores (5.3–6.2 × 3.0–3.5 μm). (Figures 2L, 8, **10F**)

Basidiomata slender (IG = 41–131). Pileus 6.0–12 mm in diameter, dirty white (2A1), brownish orange (6C4–6C5) with age, applanate to plane, surface dry, smooth. Context less than 0.5 mm thick. Lamellae 0.3–0.5 mm broad, free, subdistant, with 2–3 series of lamellulae, dirty white becoming light orange (6A4) to light brown (6C5–6D6) as mature. Stipe 13–23 × 0.3–0.5 mm, cylindrical, slender, hollow, white with yellowish gray (4B2) tinges, surface with white (1A1) fibrils. Annulus single, membranous, light brown (6C5–6D6), persistent, superior to median.

Basidiospores (5.0) 5.3–6.2 (6.5) × 3.0–3.5 μm, av. = 5.8 × 3.3 μm, Q = (1.56) 1.61–1.91 (2.07), elongated or few ellipsoid in face view, amygdaliform in profile view, light brown, wall 0.3–0.5 μm thick, with or without an apically thickened endosporium, without germ pore, inamyloid. Basidia 9.5–12 × 6.0–7.0 μm, clavate, hyaline, 4- or 2-spored. Pleurocystidia absent. Cheilocystidia 14–26 × (6.0) 8.0–11.5 (12.5) μm, hyaline, claviform or widely utriform, apex broadly obtuse, rare subcapitate, covered by hyaline deposition that dissolves in 5% KOH. Pileipellis a cutis, hyphae 6.0–15 μm in diameter, some constricted at the septa on some hyphae, with hyaline, high refractive inclusions that dissolves in 5% KOH.

Habit and habitat: Gregarious on ground in forest.

Specimens examined: CHINA. Fujian Province, Sanming Castanopsis fargesii Provincial Nature Reserve, 18 Jun 2020, Jun-Qing Yan, Hui Zeng, Sheng-Nan Wang, HFJAU1536, holotype; Wuyishan National Park, 12 Jun 2021, Jun-Qing Yan, HFJAU3178.

Notes: *Micropsalliota tenuipes* is characterized by its slender stipe compared to the size of the pileus (6.0–12 mm). *Micropsalliota arginea, M. cinnamomeopallida, M. plumeria*, and *M. pseudoarginea* resemble *M. tenuipes* but have smaller basidiospores (< 5.5 μm long) (Pegler and Rayner, 1969; Heinemann, 1980, 1988; Heinemann and Leelavathy, 1991; Zhao et al., 2010). In the phylogenetic tree, *M. tenuipes* groups together with *M. albella*, but the latter has smaller basidiomata (a pileus 2.0–5.0 mm in diameter) and basidiospores shorter than 5.5 μm (He et al., 2020).

#### Micropsalliota wuyishanensis J. Q. Yan sp. nov.

MycoBank: 842898

Etymology: Referring to its type locality (Wuyishan National Park).

**Figure 9:**
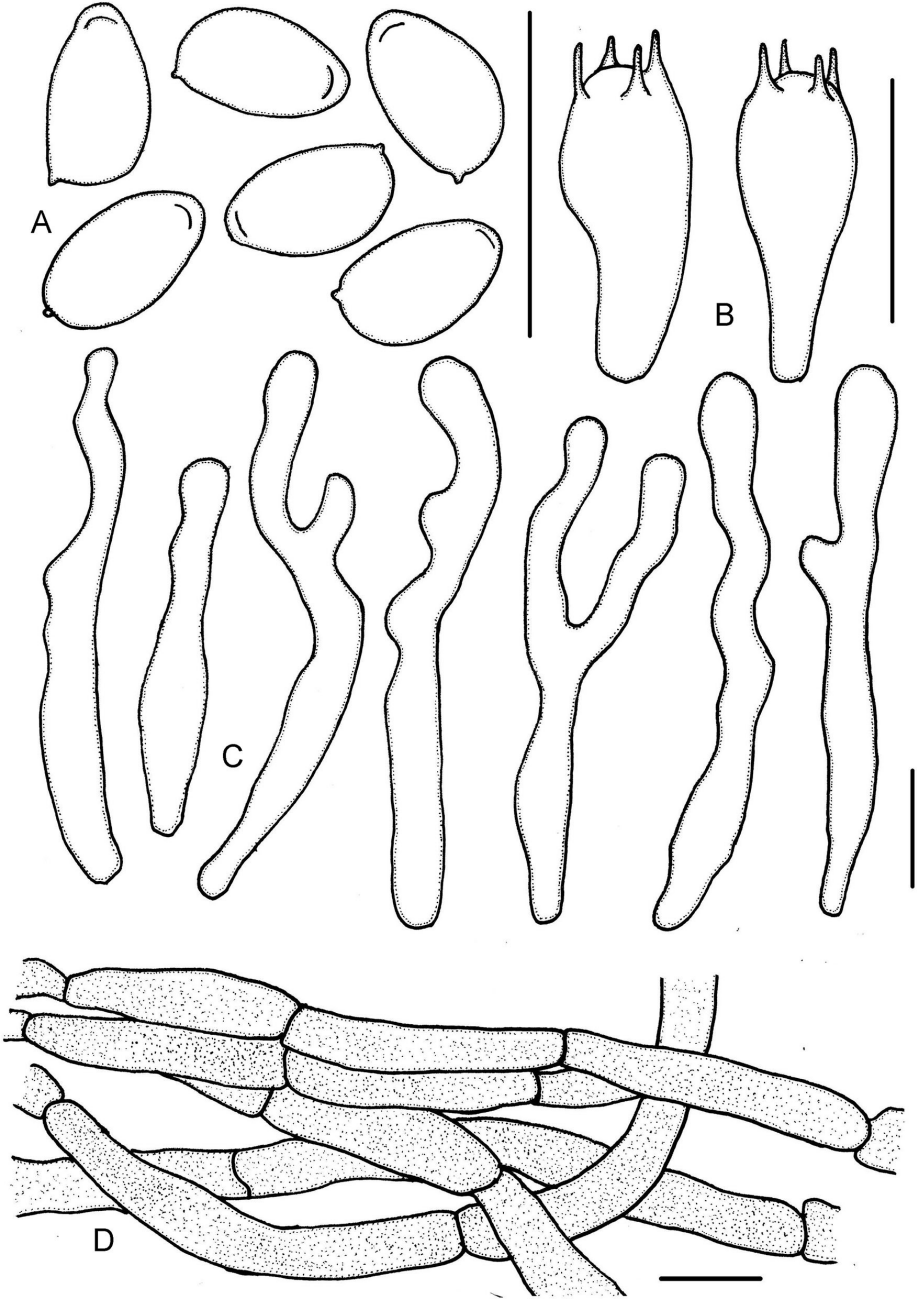
*Micropsalliota wuyishanensis*. **(A)** Basidiospores; **(B)** basidia; **(C)** cheilocystidia; **(D)** pileipellis hyphae. Scale bars: 10 μm.

**Figure 10:**
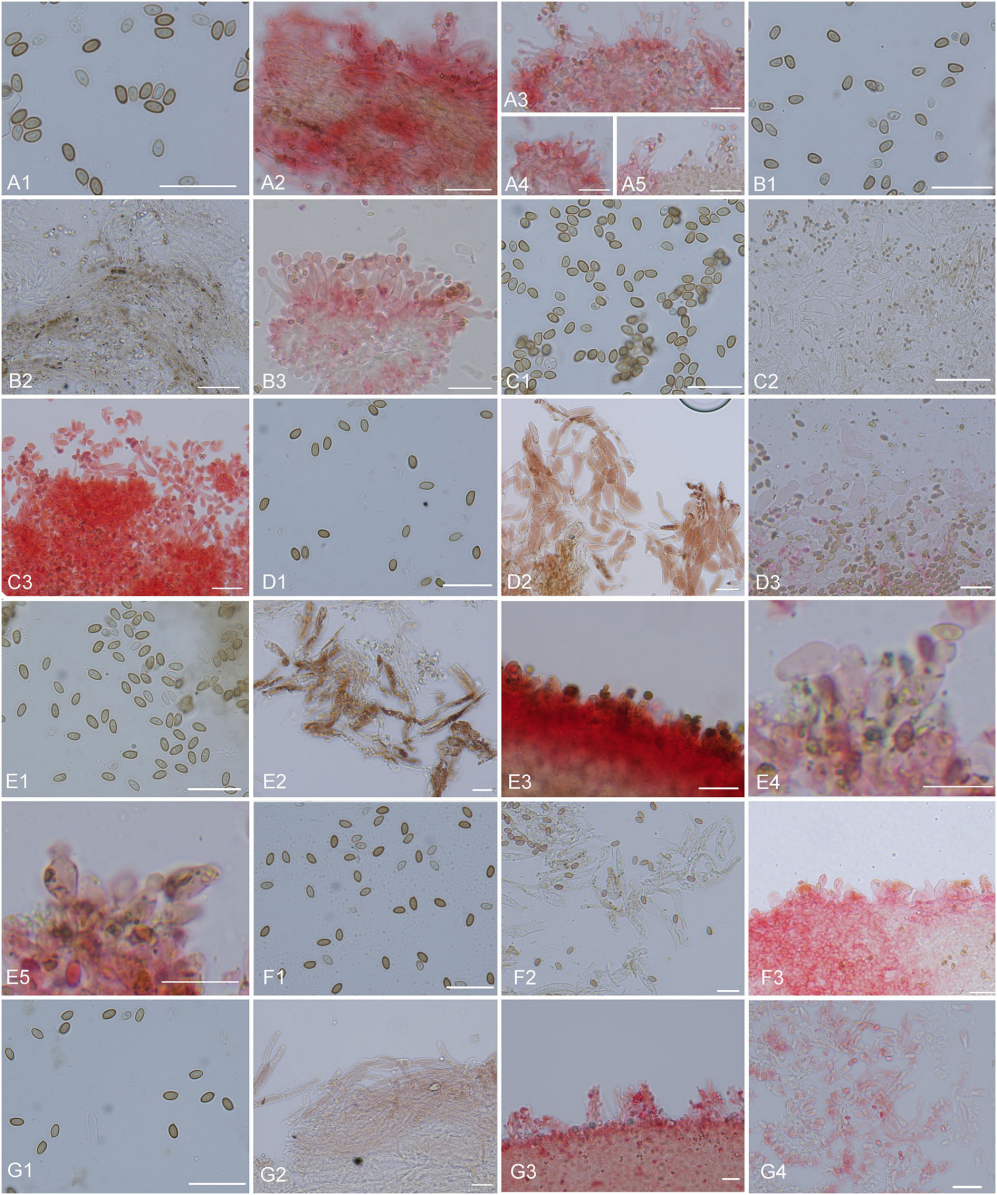
Microscopic structures. **(A)** *Micropsalliota minor*: **(A1)** basidiospores, **(A2)** pileipellis hyphae, **(A3–5)** marginal cell of gills and cheilocystidia; **(B)** *M. ovalispora*: **(B1)** basidiospores, **(B2)** pileipellis hyphae, **(B3)** marginal cell of gills and cheilocystidia; **(C)** *M. pseudodelicatula*: **(C1)** basidiospores, **(C2)** pileipellis hyphae, **(C3)** marginal cell of gills and cheilocystidia; **(D)** *M*. cf. *roseipes*: **(D1)** basidiospores, **(D2)** pileipellis hyphae, **(D3)** marginal cell of gills and cheilocystidia; **(E)** *M. rufosquarrosa*: **(E1)** basidiospores, **(E2)** pileipellis hyphae, **(E3–5)** marginal cell of gills and cheilocystidia; **(F)** *M. tenuipes*: **(F1)** basidiospores, **(F2)** pileipellis hyphae, **(F3)** marginal cell of gills and cheilocystidia; **(G)** *M. wuyishanensis*: **(G1)** basidiospores, **(G2)** pileipellis hyphae, **(G3)** marginal cell of gills and cheilocystidia. Scale bars = 20 μm. Structures of **(B2–G2)** were observed in water. Other structures were observed 5% KOH, Congo red was used as a stain when necessary.

Diagnosis: Differs from *M. cardinalis* by its stipe covered with red fibrils and by its hyphoid, often forked, cheilocystidia. (Figures 2M, 9, 10G)

Basidiomata slender to stout (IG = 28–43). Pileus 6.0–10 mm in diameter, red (10A6–10B7), campanulate, surface dry, covered with deep red (10C7–10C8) fibrils, margin appendiculate, with white or pinkish remains of the veil. Context less than 0.5 mm thick. Lamellae 0.5–1.0 mm broad, free, moderately distant, with 3 series of lamellulae, white (1A1) becoming light brown (7D5–7D6) as mature. Stipe 18–25 × 1.0–1.5 mm, cylindrical, uniform, white (1A1), surface with pastel red to dull red (10A4–10B4) fibrils. Annulus single, membranous, white (1A1), superior, fall off when young.

Basidiospores (5.0) 5.5–6.0 (6.5) × 3.0–3.5 (4.0) μm, av. = 5.8 × 3.4 μm, Q = 1.60–1.80 (1.90), elongated in face view, amygdaliform in profile view, light brown, without germ pore, inamyloid, thick-walled, with a thickened apical endosporium. Basidia (13) 14–18.5 (20) × (5.0) 6.0–7.0 (7.5) μm, clavate, hyaline, 4-spored. Pleurocystidia absent. Cheilocystidia 33–60 (72) × 3.5–6.0 (6.5) μm, hyaline, hyphoid, tortuous or submoniliform, often branched or forked, apex obtuse or subcapitate, rare capitate. Pileipellis a cutis, hyphae (4.5) 6.5–10 (11) μm in diameter, with pink membranous pigment.

Habit and habitat: Scattered on soil in broad-leaved forests.

Specimens examined: CHINA. Fujian Province, Wuyishan National Park, Jun-Qing Yan, Ya-Ping Hu and Hui Ding, 10 Aug 2021, HFJAU3048, holotype.

Notes: Among known species of *Micropsalliota*, only *M. cardinalis* and *M. roseipes* resemble *M. wuyishanensis* in having a red pileus 5.0–10 mm in diameter. These two species can be distinguished from the latter as follows: *M. cardinalis* has capitate cheilocystidia that are less than 35 μm long (Heinemann, 1989), and *M. roseipes* has longer basidiospores (6.2–7.4 μm) (Heinemann, 1980). In the phylogenetic tree, *M. wuyishanensis* formed a distinct lineage, but the phylogenetic position and relationships with other species are still unclear.

### Key to *Micropsalliota* species of China

1a. Pileus white to dirty white……………………………..2
1b. Pileus colored, e.g., brown, red or violet………………10
2a. Basidiospores shorter than 5.5 μm……………………..3
2b. Basidiospores longer than 5.5 μm………….…………..6
3a. Cheilocystidia bifid with two toe-like subcapitate lobes…………………….……………………*M. bifida*
3b. Not as above……………………………….…………4
4a. Basidiospores ovoid in face view, amygdaliform in profile view………………………….….…….….*M. ovalispora*
4b. Not as above…………………….…….………………5
5a. Pileus < 5 mm in diameter, cheilocystidia tibiiform or lageniform, apex capitate……………*M. pseudodelicatula*
5b. Pileus >5 mm in diameter, cheilocystidia non-capitate……….….………………*M. pseudoarginea*
6a. Pileus mainly < 10 mm in diameter…….….……………7
7a. Cheilocystidia two types, tibiiform or forked with capitate or subacute apex………………………………*M. minor*
7b. Not as above………….…….…….……………………8
8a. Cheilocystidia utriform, with broadly obtuse apex…….…….…….………………………*M. tenuipes*
8b. Cheilocystidia capitate, with a sinuous or straight neck………………………………………*M. delicatula*
9a. Pileipellis hyphae with vacuolar pigments….….*M. subalba*
9b. Pileipellis hyphae hyaline but with pigment incrusted……….…………………*M. dentatomarginata*
10a. Pleurocystidia present…….….…………*M. digitatocystis*
10b. Pleurocystidia absent…………………………………11
11a. Pileus mainly < 10 mm in diameter……………………12
11b. Pileus larger than 10 mm in diameter……………….…15
12a. Pileus brown to dark brown…….….………*M. megaspora*
12b. Pileus pink, red to violet-red…………………….……13
13a. Cheilocystidia hyphoid, often forked, up to 60 μm long………………….….……………*M. wuyishanensis*
13b. Not as above…………………………………………14
14a. Stipe dirty white with pink tone, cheilocystidia without deposit……………………….…….….…*M*. cf. *roseipes*
14b. Stipe white, cheilocystidia covered by light brown deposit………………………………..*M. rufosquarrosa*
15a. Pileus 20–80 mm in diameter…………………………16
15b. Pileus < 20 mm in diameter…………………………...18
16a. Basidiospores 4.5–6 μm long………..*M. pseudoglobocystis*
16b. Basidiospores longer than 6 μm………………………17
17a. Conext stains red when bruised or cut………………………………………………………..*M. furfuracea*
17b. Conext stains yellow to reddish-brown…………………………………………………………..*M. globocystis*
18a. Veil cortinate………………………………*M. cortinata*
18b. Veil membranous……………………………………19
19a. Basidiomes violet-red…………*M. lateritia* var. *vinaceipes*
19b. Basidiomes brown………………………*M. arginophaea*

## Discussions

The results of our phylogenetic analysis are to some extent consistent with previous studies based on ITS and LSU sequences (Zhao et al., 2010; He et al., 2020; Li et al., 2021; Al-Kharousi et al., 2022), but we were able to resolve more subclades, and the support values for some clades and subclades were higher. Major clades were strongly to weakly supported, but were difficult to morphologically characterize. Many comprised a morphologically heterogeneous assemblage of species, with only a few subclades that were synapomorphies (see below).

In clade A, species in /megaspora share fibrillose to floccose, brown to dark brown squamules, an IG < 50, and basidiospores up to 7.5 μm long (Heinemann, 1980; Zhao et al., 2010). Among /pleurocystidiata, *M. pleurocystidiata* and *M. xanthorubescens* share fibrillose to floccose, brown to dark brown squamules, presence of pleurocystidia, basidiospores 5.0–7.0 μm long, and an IG < 30 (Heinemann, 1980; Heinemann and Flower, 1983; Zhao et al., 2010).

In Clade B, species in /rufosquarrosa share red squamules, basidiospores 5.5–7.0 μm long, and an IG < 30 (Heinemann, 1980). Species in /bifida, except for *M. arginophaea* Heinem. and *M. gracilis* Heinem., have white and squamulose pileus, but there are no obvious synapomorphies in regard to the basidiospore size, IG value, or color change upon bruising (Heinemann and Flower, 1983; Zhao et al., 2010; He et al., 2020; Li et al., 2021). Species in /allantoidea share small basidiomata, a pileus less than 10 mm in diameter, capitate cheilocystidia with an elongated neck, and an IG = 30–60 (Zhao et al., 2010); species in /*arginea* are not obviously morphologically synapomorphies except in having spores that are ellipsoid to amygdaliform, which is true for most species of *Micropsalliota* (Pegler and Rayner, 1969; Zhao et al., 2010; Chen et al., 2016; Parra et al., 2016).

Species in Clade D share fibrillose or squamulose pileus and mainly basidiospores longer than 6 μm, but this common feature also applies to Clades A, E, and a few species in area F. Therefore, this feature is not representative. In addition, except for *M. megarubescens*, most of the species in this clade resemble *M. globocystis* in their macroscopic features: they have brown to red-brown squamules, basidiospores mainly shorter than 7.5 μm, and an IG < 30 (Zhao et al., 2010; Wei et al., 2015; He et al., 2020; Li et al., 2021).

Clade C is characterized by the presence of a cortinoid annulus (Heinemann, 1980; Zhao et al., 2010). Clade E share moderately sized basidiomes (IG = 40–80) with a white, silky pileus that stains reddish brown (Zhao et al., 2010). Although area F was undetermined, species in this area, except for *M. ventricocystidiata*, share red to red-brown squamules, an IG < 40, basidiospores mainly shorter than 6 μm, and more or less forked cheilocystidia (Zhao et al., 2010; Al-Kharousi et al., 2022).

Based on the study of Al-Kharousi et al. (2022), species of *Micropsalliota* recovered in two groups: M-I and M-II. Clade M-I shares fibrillose/squamulose pileus and the average length of basidiospores is ≥ 6.0 μm, which contains the majority of species in the Clade A, C, D, E, and area F, but *M. wuyishanensis* does not conform to this characteristic combination (this paper). Clade M-II shares more or less glabrous pileus and spores are < 6.0 μm in length by phylogenetic analyses, which contains the majority of species in the /bifida and /allantoidea, but *M. subalba* Heinem. and Little Flower does not conform to this characteristic combination (this paper).

The species diversity of this genus may be much richer than we previously thought. As more species are discovered, we believe that more well-supported clades will appear in the phylogenetic tree and synapomorphies will be better resolved. Beyond that, some seemingly known species deserve further study: the three sequences of *M. subarginea* from Thailand do not form a distinct lineage and may thus represent three separate species; and the sequences of *M. globocystis* from China and Thailand form five clades and may represent five different species. Some names may be synonyms: although phylogenetic placement suggests that *M. pleurocystidiata* and *M. xanthorubescens* are conspecific, taking into account that the type specimens have not been sequenced, we tentatively maintain them as independent species in accordance with the treatment of Zhao et al. (2010); *M. cornuta* is thus very likely a synonym of *A. trisulphuratus*; however, we have not examined the type specimen of *A. trisulphuratus* and cannot draw definite conclusions.

## Data availability statement

The datasets presented in this study can be found in online repositories. The names of the repository/repositories and accession number(s) can be found in the article/Supplementary material.

## Author contributions

J-QY and S-NW designed the research and contributed to data analysis and interpretation. Z-HZ, Y-PH, B-RK, and HZ participated in the collection of specimens. All authors contributed to the article and approved the submitted version.

## Funding

This work was supported by the National Natural Science Foundation of China (31960008), the Project of FAAS (XTCXGC2021007), Biodiversity Investigation, Observation and Assessment Program (2019–2023) of the Ministry of Ecology and Environment of China (2110404), Project of Biological Resources Survey in Wuyishan National Park (HXQT2020120701), and Project of Biodiversity Conservation in Lishui, Zhejiang Province (HXYJCP2021110648).

## Conflict of interest

The authors declare that the research was conducted in the absence of any commercial or financial relationships that could be construed as a potential conflict of interest.

## Publisher's note

All claims expressed in this article are solely those of the authors and do not necessarily represent those of their affiliated organizations, or those of the publisher, the editors and the reviewers. Any product that may be evaluated in this article, or claim that may be made by its manufacturer, is not guaranteed or endorsed by the publisher.

## References

[B1] Al-Kharousi M. Hussain S. Al-Muharabi M. A. Al-Shabibi Z. Al-Balushi A. H. Al-Yahya'ei M. N.. (2022). Notes on the genus *Micropsalliota* (Agaricales, Basidiomycota) and the description of a new species from Southern Oman. Phytotaxa 543, 113–126. 10.11646/phytotaxa.543.2.2

[B2] Bas C. (1969). Morphology and subdivision of *Amanita* and a monograph of its section *Lepidella*. Persoonia 5, 285–573.

[B3] Bau T. Yan J.-Q. (2021). A new genus and four new species in the /Psathyrella s.l. clade from China. MycoKeys 80, 115–131. 10.3897/mycokeys.80.65123 34131386 PMC8172522

[B4] Chen J. Hyde K. D. Bahkali A. H. Zhao R. L. (2016). *Micropsalliota brunneosquamata*, a new species from Thailand. Chiang Mai J. Sci. 43, 689–694.34899100

[B5] Chen Y. L. Pei N. C. Zhang L. P. Deng W. Q. Wu F. Liu M. (2019). Three new records of *Micropsalliota* from China. Acta Agric. Univ. Jiangxiensis 41, 708–714. 10.13836/j.jjau.201908234899100

[B6] Crous P. Osieck E. Jurjevi Ž. Boers J. Van Iperen A. Starink-Willemse M.. (2021). Fungal planet description sheets: 1284–1382. Persoonia Mol. Phyl. Evol. Fungi 47, 178–374. 10.3767/persoonia.2021.47.06 PMC10486635 37693795

[B7] Ge Y. P. Liu Z. W. Zeng H. Cheng X. H. Na Q. (2021). Updated description of *Atheniella* (Mycenaceae, Agaricales), including three new species with brightly coloured pilei from Yunnan Province, southwest China. MycoKeys 81, 139–164. 10.3897/mycokeys.81.67773 34305422 PMC8295246

[B8] Guzmán-Dávalos L. (1992). First record of the genus *Micropsalliota* (Basidiomycotina, Agaricaceae) in Mexico. Mycotaxon 43, 199–205.

[B9] Guzmán-Dávalos L. Heinemann P. (1994). *Micropsalliota subalpina* nov. sp. (Agaricaceae) from Mexico. Bull. Jardin Bot. Natl. Belg. 63, 195–199. 10.2307/3668476

[B10] He M. Q. Hyde K. D. Cheewangkoon R. Zhao R. L. (2020). Two new species of *Micropsalliota* (Agaricaceae/Agaricales) from Thailand. Phytotaxa 453, 137–144. 10.11646/phytotaxa.453.2.5

[B11] Heinemann P. (1956). Champignons récoltés au Congo Belge par Madame M. Goossens-Fontana II. *Agaricus* Fries ss. Bull. Jardin Bot. Natl. Belg. 26, 1–127. 10.2307/3667096

[B12] Heinemann P. (1977). The genus *Micropsalliota*. Kew Bull. 31, 581–583. 10.2307/4119406

[B13] Heinemann P. (1980). Les genres *Agaricus* et *Micropsalliota* en Malaisie et en Indonésie. Bull. Jardin Bot. Natl. Belg. 50, 3–68. 10.2307/3667774

[B14] Heinemann P. (1983). Clé de détermination de *Micropsalliota* (Agaricaceae) et description de deux espèces nouvelles. Bull. Jardin Bot. Natl. Belg. 53, 85–95. 10.2307/3668030

[B15] Heinemann P. (1988). Novitates generis *Micropsalliotae* (Agaricaceae). Bull. Jardin Bot. Natl. Belg. 58, 540–543. 10.2307/3668305

[B16] Heinemann P. (1989). Le genre *Micropsalliota* en Amérique tropicale et subtropicale. Bull. Jardin Bot. Natl. Belg. 59, 459–466. 10.2307/3668360

[B17] Heinemann P. Flower S. L. (1983). Micropsalliota de Kerala (Inde). Bull. Jardin Bot. Natl. Belg. 53, 75–84. 10.2307/3668029

[B18] Heinemann P. Leelavathy K. M. (1991). The genus *Micropsalliota* (Agaricaceae) in Kerala State, India. Mycol. Res. 95, 341–346. 10.1016/S0953-7562(09)81245-8

[B19] Höhnel F. X. R. (1914). Fragmente zur Mykologie XVI (XVI. Mitteilung, Nr. 813 bis 875). Sitzungsberichte der Kaiserlichen Akademie der Wissenschaften Math.-naturw. Klasse Abt. I 123, 49–155.

[B20] Hopple J. J. Vilgalys R. (1999). Phylogenetic relationships in the mushroom genus *Coprinus* and dark-spored allies based on sequence data from the nuclear gene coding for the large ribosomal subunit RNA: divergent domains, outgroups, and monophyly. Mol. Phylogenet. Evol. 13, 1–19. 10.1006/mpev.1999.0634 10508535

[B21] Horak E. (2005). Röhrlinge und Blätterpilze in Europa: Bestimmungsschlüssel für Polyporales (pp), Boletales, Agaricales, Russulales. Munich: Elsevier.

[B22] Katoh K. Rozewicki J. Yamada K. D. (2019). MAFFT online service: multiple sequence alignment, interactive sequence choice and visualization. Brief Bioinformat. 20, 1160–1166. 10.1093/bib/bbx108 28968734 PMC6781576

[B23] Kornerup A. Wanscher J. H. K. (1978). The Methuen Handbook of Colour. 3rd Edn. London: Eyre Methuen Ltd. Reprint.

[B24] Lanfear R. Frandsen P. B. Wright A. M. Senfeld T. Calcott B. (2017). PartitionFinder 2: new methods for selecting partitioned models of evolution for molecular and morphological phylogenetic analyses. Mol. Biol. Evol. 34, 772–773. 10.1093/molbev/msw260 28013191

[B25] Li J. X. He M. Q. Zhao R. L. (2021). Three new species of *Micropsalliota* (Agaricaceae, Agaricales) from China. Phytotaxa 491, 167–176. 10.11646/phytotaxa.491.2.6

[B26] Li Y. Li T. H. Yang Z. L. Bau T. Dai Y. C. (2015). Altas of Chinese Macrofungal Resources. Henan: Central Plains Farmers Publishing House, China.

[B27] Liu W. Chen Y. L. Zhang L. P. Lliang J. F. (2021). Two new records of the genus *Micropsalliota* from China. Chin. J. Trop. Crops 43, 703–709. 10.3969/j.issn.1000-2561.2022.04.006

[B28] Matheny P. B. (2005). Improving phylogenetic inference of mushrooms with RPB1 and RPB2 nucleotide sequences (*Inocybe*; Agaricales). Mol. Phylogenet. Evol. 35, 1–20. 10.1016/j.ympev.2004.11.014 15737578

[B29] Na Q. Liu Z. W. Zeng H. Cheng X. H. Ge Y. P. (2022). Crepidotus yuanchui sp. nov. and *C. caspari found in subalpine areas of China. Mycoscience* 63, 1–11. 10.47371/mycosci.2021.10.004 PMC10043943 37091218

[B30] Nguyen L. T. Schmidt H. A. von Haeseler A. Minh B. Q. (2014). IQ-TREE: a fast and effective stochastic algorithm for estimating maximum-likelihood phylogenies. Mol. Biol. Evol. 32, 268–274. 10.1093/molbev/msu300 25371430 PMC4271533

[B31] Parra L. A. Tan Y. Xu M. L. Zhou J. L. Wang B. Zhao R. L. (2016). A reexamination of *Allopsalliota indicates* synonymy with *Micropsalliota* (Agariceae, Agaricaceae, Agaricales, basidiomycota). Mycoscience 57, 303–310. 10.1016/j.myc.2016.02.005

[B32] Pegler D. N. Rayner R. W. (1969). A contribution to the agaric flora of Kenya. Kew Bull. 23, 347–412. 10.2307/4117177

[B33] Ronquist F. Teslenko M. Van Der Mark P. Ayres D. L. Darling A. Höhna S.. (2012). MrBayes 3.2: efficient Bayesian phylogenetic inference and model choice across a large model space. Syst. Biol. 61, 539–542. 10.1093/sysbio/sys029 22357727 PMC3329765

[B34] Singer R. (1975). The Agaricales in Modern Taxonomy. Vaduz: J. Cramer.

[B35] Sun J. Y. Wang Y. S. Wang X. Wang Y. Yi Z. C. Yan J. Q.. (2020). Two new records of *Micropsalliota* from Jiangxi Province. J. Northeast For. Univ. 48, 120–123.

[B36] Wang F. J. Zhou X. Y. Gan L. (2017). *Micropsalliota lateritia*, a new record of Micropsalliota from Hubei Province in China. J. Central China Normal Univ. 51, 651–654. 10.19603/j.cnki.1000-1190.2017.05.016

[B37] Wang S. N. Fan Y. G. Yan J. Q. (2022). Iugisporipsathyra reticulopilea gen. et sp. nov. (Agaricales, Psathyrellaceae) from tropical China produces unique ridge-ornamented spores with an obvious suprahilar plage. MycoKeys 90, 147–162. 10.3897/mycokeys.90.85690 PMC9849077 36760424

[B38] Wei L. Li Y. H. Hyde K. D. Zhao R. L. (2015). *Micropsalliota pseudoglobocystis*, a new species from China. Mycotaxon 130, 555–561. 10.5248/130.555

[B39] White T. J. Bruns T. D. Lee S. B. Taylor J. W. Innis M. A. Gelfand D. H.. (1990). Amplification and direct sequencing of Fungal Ribosomal RNA Genes for phylogenetics. San Diego: Academic Press.

[B40] Yu W. J. Chang C. Qin L. W. Zeng N. K. Wang S. X. Fan Y. G. (2020). *Pseudosperma citrinostipes* (Inocybaceae), a new species associated with Keteleeria from southwestern China. Phytotaxa 450, 8–16. 10.11646/phytotaxa.450.1.2

[B41] Zhao R. L. Desjardin D. E. Soytong K. Perry B. A. Hyde K. D. (2010). A monograph of *Micropsalliota* in Northern Thailand based on morphological and molecular data. Fungal Divers. 45, 33–79. 10.1007/s13225-010-0050-4

