# Supplementary material for: Taxonomy and multi-gene phylogeny of Micropsalliota (Agaricales, Agaricaceae) with description of six new species from China
Source: Front Microbiol. 2022 Nov 7;13:1011794. doi: 10.3389/fmicb.2022.1011794 (PMC9676474; doi:10.3389/fmicb.2022.1011794)
Supplement: Supplementary file 1 [file Table_1.DOCX]

Supplementary Material 1

TABLE S1 | Taxon sampling information and DNA sequences used for phylogenetic analyses.

| **Taxa** | **Origin** | **Specimen number** | **ITS** | **LSU** | ***rpb2*** |
| --- | --- | --- | --- | --- | --- |
| *M. albella* M.Q. He & R.L. Zhao | Thailand | MFLUCC17_1342 Type | MN294514 | MN294516 | — |
| *M. albofelina* D.D. Ivanova & O.V. Morozova | Vietnam | LE312536 Type | OK257212 | OK257209 | — |
| *M. albosericea* Heinem. & Leelav. | Thailand | ZRL3049 | HM436644 | — | — |
| *M. allantoidea* (Berk. & Broome) Pegler & R.W. Rayner | Thailand | ZRL2038 Type | HM436648 | HM436597 | — |
| *M. arginea* (Berk. & Broome) Pegler & R.W. Rayner | Thailand | ZRL3090 | — | HM436595 | — |
| *M. arginophaea* Heinem. | China (Guangxi) | GX20170167 | MT671226 | MT671244 | — |
| *M. arginophaea* | China (Jiangxi) | JXSB1685 | MK402219 | MK402227 | — |
| *M. arginophaea* | Thailand | ZRL2027 | HM436614 | HM436579 | — |
| *M. arginophaea* | Thailand | ZRL2088 | HM436615 | — | — |
| *M. arginophaea* | Thailand | ZRL2089 | HM436616 | HM436578 | — |
| *M. arginophaea* | Thailand | ZRL2091 | HM436613 | HM436580 | — |
| *M. arginophaea* | Thailand | ZRL3110 | HM436617 | HM436577 | — |
| *M. bifida* R.L. Zhao, Desjardin, Soytong & K.D. Hyde | Thailand | ZRL2057 | HM436642 | — | — |
| *M. bifida* | Thailand | ZRL2103 | HM436641 | — | — |
| *M. bifida* | Thailand | ZRL3067 Type | HM436640 | HM436591 | — |
| *M. bifida* | Thailand | ZRL3076 | HM436639 | HM436592 | — |
| *M. bifida* | Thailand | ecv3638 | HM488762 | HM488768 | HM488794 |
| *M. bifida* | China (Guangdong) | HFJAU1348 | **OM650271** | **OM650251** | **OM669857** |
| *M. bifida* | China (Fujian) | HFJAU2998 | **OM650272** | **OM650252** | **OM669858** |
| *M. brunneosquamata* Linda J. Chen, R.L. Zhao & K.D. Hyde | Thailand | LD201236 Type | KP316210 | — | — |
| *M. cornuta* Har. Takah. & Taneyama | Japan | TNS_F_52279 Type | AB968240 | — | — |
| *M. cortinata* (Heinem.) Heinem. | Thailand | ZRL2129 | HM436630 | HM436593 | — |
| *M. cortinata* | China (Jiangxi) | HFJAU0713 | MN508426 | **OM650253** | **OM669874** |
| *M. delicatula* R.L. Zhao, J.X. Li & M.Q. He | China (Zhejiang) | HMAS290752 Type | MT671229 | — | — |
| *M. delicatula* | China (Zhejiang) | HMAS290753 | MT671230 | MT671243 | — |
| *M. dentatomarginata* R.L. Zhao, J.X. Li & M.Q. He | China (Guangxi) | HMAS290760 Type | MT671228 | MT671242 | — |
| *M. digitatocystis* R.L. Zhao, J.X. Li & M.Q. He | China (Guangxi) | HMAS290751 | MT671238 | MT671249 | — |
| *M. digitatocystis* | China (Yunnan) | HMAS290750 Type | MT671239 | MT671250 | — |
| *M. digitatocystis* | China (Jiangxi) | HFJAU1871 | **OM650273** | — | **OM669855** |
| *M. furfuracea* R.L. Zhao, Desjardin, Soytong & K.D. Hyde | China (Zhejiang) | HFJAU1768 | **OM650274** | — | **OM669871** |
| *M. furfuracea* | China | K15091214 | MH045826 | — | — |
| *M. furfuracea* | China | K15091228 | MH045827 | — | — |
| *M. furfuracea* | China (Guangdong) | GDGM44176 | KJ849235 | — | — |
| *M. furfuracea* | China (Hainan) | N.K.Zeng2420 | MT822910 | — | — |
| *M. furfuracea* | Thailand | ZRL2119 | HM436622 | HM436604 | — |
| *M. furfuracea* | Thailand | ZRL3006 Type | HM436621 | HM436603 | — |
| *M. furfuracea* | China (Jiangxi) | HFJAU1570 | **OM650275** | — | **OM669872** |
| *M. furfuracea* | China (Fujian) | HFJAU3123 | **OM650276** | **OM650254** | **OM669873** |
| *M. geesterani* (Bas & Heinem.) R.L. Zhao & L.A. Parra | Thailand | AH47609 | KM923965 | KM923966 | — |
| *M. geesterani* | The Netherlands | E.C. Vellinga 2263(L) | AF482857 | AF482888 | — |
| *M. globocystis* Heinem. | China (Zhejiang) | HFJAU2709 | **OM650278** | **OM650262** | **OM669856** |
| *M. globocystis* | China | H15060610 | MH045830 | — | — |
| *M. globocystis* | China | H16091312 | MH045831 | — | — |
| *M. globocystis* | China (Jiangxi) | JXSB819 | MK402215 | MK402223 | — |
| *M. globocystis* | China | K15091213 | MH045832 | — | — |
| *M. globocystis* | China | K16053117 | MH045833 | — | — |
| *M. globocystis* | China | ZRL2013465 | LT716024 | KY418839 | KY418991 |
| *M. globocystis* | China (Hunan) | MHHNU31093 | MK239247 | — | — |
| *M. globocystis* | China (Zhejiang) | ZRL2015164 | MT671236 | MT671251 | — |
| *M. globocystis* | China (Zhejiang) | ZRL2015243 | MT671237 | MT671252 | — |
| *M. globocystis* | Laos | HNL501440 | MW073388 | — | — |
| *M. globocystis* | Laos | HNL501777 | MW073389 | — | — |
| *M. globocystis* | South Africa | VDW1278 | MT304640 | — | — |
| *M. globocystis* | Thailand | ZRL2049 | HM436635 | — | — |
| *M. globocystis* | Thailand | ZRL2133 | HM436632 | — | — |
| *M. globocystis* | China (Fujian) | HFJAU1518 | **OM650277** | **OM650255** | **OM669852** |
| *M. globocystis* | Thailand | ZRL2126 | HM436633 | — | — |
| *M. globocystis* | Thailand | ZRL3004 | HM436634 | HM436605 | — |
| *M. gracilis* Heinem. | Laos | HNL503432 | MW192914 | — | — |
| *M. gracilis* | Laos | HNL503436 | MW192915 | — | — |
| *M. gracilis* | Thailand | NW1343 | MW426435 | — | — |
| *M. gracilis* | Thailand | ZRL2041 | HM436647 | HM436583 | — |
| *M. lateritia* var. *vinaceipes* R.L. Zhao, Desjardin, Soytong & K.D. Hyde | China (Hubei) | HMHNU950 | MH155967 | — | — |
| *M. lateritia* var. *vinaceipes* | China (Hubei) | HMHNU930 | KY779732 | — | — |
| *M. lateritia* var. *vinaceipes* | Thailand | ZRL2073 Type | HM436631 | — | — |
| *M. lateritia* var. *vinaceipes* | China (Jiangxi) | HFJAU1885 | **OM650279** | — | **OM669877** |
| *M. megarubescens* R.L. Zhao, Desjardin, Soytong & K.D. Hyde | China (Hainan) | N.K.Zeng2989 | MT822911 | — | — |
| *M. megarubescens* | China (Zhejiang) | HMAS290754 | MT671235 | MT671247 | — |
| *M. megarubescens* | Thailand | ZRL2008 | HM436618 | HM436602 | — |
| *M. megarubescens* | Thailand | ZRL2009 | HM436619 | HM436601 | — |
| *M. megarubescens* | Thailand | ZRL2086 Type | HM436620 | — | — |
| *M. megaspora* R.L. Zhao, Desjardin, Soytong & K.D. Hyde 2010 | China (Jiangxi) | HFJAU0712 | **OM650280** | **OM650256** | **OM669875** |
| *M. megaspora* | China (Jiangxi) | HFJAU1209 | **OM650281** | **OM650257** | — |
| *M. megaspora* | Thailand | ZRL2051 | HM436623 | HM436571 | — |
| *M. megaspora* | Thailand | ZRL3068 | HM436624 | HM436572 | — |
| *M. megaspora* | China (Jiangxi) | HFJAU1255 | **OM650282** | **OM650258** | **OM669876** |
| *M. minor* J.Q. Yan | China (Zhejiang) | HFJAU2796 | **OM650294** | **OM650266** | **OM669865** |
| *M. minor* | China (Zhejiang) | HFJAU2812 Type | **OM650293** | — | **OM669864** |
| *M. ovalispora* J.Q. Yan | China (Zhejiang) | HFJAU2010 Type | **OM650295** | **OM650269** | **OM669866** |
| *M. ovalispora* | China (Zhejiang) | HFJAU3179 | **OM650296** | — | **OM669867** |
| *M. pleurocystidiata* Heinem. & Little Flower | Thailand | ZRL2023 | HM436636 | — | — |
| *M. pseudoarginea* Heinem. | China (Zhejiang) | HFJAU1715 | **OM650283** | **OM650259** | **OM669859** |
| *M. pseudoarginea* | China (Zhejiang) | HFJAU2122 | **OM650284** | **OM650260** | **OM669861** |
| *M. pseudoarginea* | China (Jiangxi) | JXSB1681 | MK402217 | MK402225 | — |
| *M. pseudoarginea* | Thailand | ZRL3069 | HM436643 | — | — |
| *M. pseudoarginea* | China (Zhejiang) | HFJAU2224 | **OM650285** | **OM650261** | **OM669860** |
| *M. pseudodelicatula* J.Q. Yan | China (Jiangxi) | HFJAU1291 | MN622758 | **OM650263** | **OM669862** |
| *M. pseudodelicatula* | China (Zhejiang) | HFJAU2228 Type | **OM650288** | **OM650264** | **OM669863** |
| *M. pseudoglobocystis* Li Wei & R.L. Zhao | China (Jiangxi) | HFJAU1556 | **OM650286** | — | **OM669853** |
| *M. pseudoglobocystis* | China (Fujian) | HFJAU2433 | **OM650287** | — | **OM669854** |
| *M. pseudoglobocystis* | China (Guangxi) | GX20172228 | MT671233 | MT671245 | — |
| *M. pseudoglobocystis* | China (Guangxi) | GX20172236 | MT671234 | MT671246 | — |
| *M. pseudoglobocystis* | China (Yunnan) | ZRL2013323 | KM889912 | — | — |
| *M. pseudoglobocystis* | China (Yunnan) | ZRL2013335 | KM889911 | — | — |
| *M. pseudoglobocystis* | Thailand | ZRL201332 Type | KM889913 | — | — |
| *M. purpureobrunneola* M.Q. He & R.L. Zhao | Thailand | MFLUCC17_1343 Type | MN294513 | MN294517 | — |
| *M. pusillissima* R.L. Zhao, Desjardin, Soytong & K.D. Hyde | Thailand | ZRL3047 Type | HM436645 | HM436594 | — |
| *M. repanda* Heinem. | Togo | AH47613 | KP739805 | KP739804 | — |
| *M.* cf. *roseipes* | China (Fujian) | HFJAU2494 | **OM650297** | **OM650270** | **OM669870** |
| *M. rubrobrunnescens* R.L. Zhao, Desjardin, Soytong & K.D. Hyde | China | K15091208 | MH045828 | — | — |
| *M. rubrobrunnescens* | China | K16082504 | MH045829 | — | — |
| *M. rubrobrunnescens* | Thailand | NW1510 | MW504979 | — | — |
| *M. rubrobrunnescens* | Thailand | NW1522 | MW504980 | — | — |
| *M. rubrobrunnescens* | Thailand | NW1523 | MW504981 | — | — |
| *M. rubrobrunnescens* | Thailand | ZRL2094 | HM436625 | HM436585 | — |
| *M. rubrobrunnescens* | Thailand | ZRL2120 Type | HM436628 | HM436588 | — |
| *M. rubrobrunnescens* | Thailand | ZRL3010 | HM436626 | HM436587 | — |
| *M. rubrobrunnescens* | Thailand | ZRL3048 | HM436627 | HM436586 | — |
| *M. rubrobrunnescens* var. *tibiicystis* R.L. Zhao, Desjardin, Soytong & K.D. Hyde | China (Guangxi) | HMAS290755 | MT671231 | MT671240 | — |
| *M. rubrobrunnescens* var. *tibiicystis* | Thailand | ZRL2121 Type | HM436629 | HM436589 | — |
| *M. rufosquarrosa* J.Q. Yan | China (Jiangxi) | HFJAU1208 | **OM650291** | **OM650267** | **OM669868** |
| *M. rufosquarrosa* | China (Jiangxi) | HFJAU1236 Type | **OM650292** | **OM650268** | **OM669869** |
| *M. subalba* Heinem. & Little Flower | Thailand | ZRL2080 | HM436646 | HM436596 | — |
| *M. subarginea* Heinem. | Thailand | ZRL2052 | HM436612 | HM436573 | — |
| *M. subarginea* | Thailand | ZRL2092 | HM436611 | HM436574 | — |
| *M. subarginea* | Thailand | ZRL3036 | HM436610 | HM436575 | — |
| *M. suthepensis* R.L. Zhao, Desjardin, Soytong & K.D. Hyde | Thailand | ZRL3035 Type | — | HM436584 | — |
| *M. tenuipes* J.Q. Yan | China (Fujian) | HFJAU1536 Type | **OM650289** | — | — |
| *M. tenuipes* | China (Fujian) | HFJAU3180 | **OM650290** | **OM650265** | — |
| *M. ventricocystidiata* Al-Sadi & S. Hussain | Oman | SQUH-ATR004 | OM397373 | OM630413 | — |
| *M. ventricocystidiata* | Oman | SQUH-GOB002 Type | OM397374 | OM630414 | — |
| *M. wuyishanensis* J.Q. Yan | China (Fujian) | HFJAU3048 Type | **OM650298** | — | **OM669878** |
| *M. xanthorubescens* Heinem. | Thailand | NW1356 | MW504965 | — | — |
| *M. xanthorubescens* | Thailand | NW1526 | MW504982 | — | — |
| *M. xanthorubescens* | Thailand | ZRL3083 | HM436638 | HM436598 | — |
| *M. xanthorubescens* | Thailand | ZRL3096 | HM436637 | HM436599 | — |
| *M.* sp. | India | HATFDI4_68 | KU752329 | — | — |
| *M.* sp. | India | SV63 | MG917970 | — | — |
| *M.* sp. | China | LD2021_6_12 | OK643823 | OK643697 | — |
| *M.* sp. | China | LD2021_6_10 | OK643822 | OK643696 | — |
| *M.* sp. | China | HBL2021_6_10 | OK643804 | OK643666 | — |
| *M.* sp. | China (Guangxi) | GX20170214 | MT671227 | MT671241 | — |
| *M.* sp. | China (Guangxi) | GX20170533 | MT671232 | MT671248 | — |
| **Outgroup** |  |  |  |  |  |
| *Agaricus crassisquamosus* R.L. Zhao | China | ZRL2012607 Type | KT951376 | KT951510 | — |
| *A. trisulphuratus* Berk. | Togo | LAPAF7 | KM657924 | KR006605 | — |
| *A. trisulphuratus* | Thailand | ZRL2128 | JN664955 | — | — |
| *A. variicystis* Linda J. Chen, K.D. Hyde & R.L. Zhao | Thailand | LD201234 Type | KT951339 | KT951517 | — |
| *Hymenagaricus epipastus* (Berk. & Broome) Heinem. & Little Flower | Thailand | ZRL3045 | HM436649 | HM436609 | — |
| *H.* sp. | Thailand | ZRL3103 | KM982450 | KM982452 | — |

Note: Newly generated sequences are in bold.
